# Intracortical myelination in musicians with absolute pitch: Quantitative morphometry using 7‐T MRI

**DOI:** 10.1002/hbm.23254

**Published:** 2016-05-10

**Authors:** Seung‐Goo Kim, Thomas R. Knösche

**Affiliations:** ^1^ Research Group for MEG and EEG—Cortical Networks and Cognitive Functions, Max Planck Institute for Human Cognitive and Brain Sciences Leipzig Germany

**Keywords:** relaxometry, planum polare, myeloarchitecture, pitch chroma, frequency discrimination, ultra‐high field magnetic resonance imaging

## Abstract

Absolute pitch (AP) is known as the ability to recognize and label the pitch chroma of a given tone without external reference. Known brain structures and functions related to AP are mainly of macroscopic aspects. To shed light on the underlying neural mechanism of AP, we investigated the intracortical myeloarchitecture in musicians with and without AP using the quantitative mapping of the longitudinal relaxation rates with ultra‐high‐field magnetic resonance imaging at 7 T. We found greater intracortical myelination for AP musicians in the anterior region of the supratemporal plane, particularly the medial region of the right planum polare (PP). In the same region of the right PP, we also found a positive correlation with a behavioral index of AP performance. In addition, we found a positive correlation with a frequency discrimination threshold in the anterolateral Heschl's gyrus in the right hemisphere, demonstrating distinctive neural processes of absolute recognition and relative discrimination of pitch. Regarding possible effects of local myelination in the cortex and the known importance of the anterior superior temporal gyrus/sulcus for the identification of auditory objects, we argue that pitch chroma may be processed as an identifiable object property in AP musicians. *Hum Brain Mapp 37:3486–3501, 2016*. © **2016 Wiley Periodicals, Inc.**

## INTRODUCTION

### Behavioral Characteristics of AP

Absolute pitch (AP) is known as the ability to recognize and label the pitch chroma of a given tone without external reference [Miyazaki, 2004a]. Behaviorally, AP is characterized by accuracy, rapidness, spontaneity, and uncontrollability. For accuracy, average hit rates were reported between 75% to 94% depending on the timbres and pitch classes of given tones, as well as on other factors such as ethnicity [Bermudez and Zatorre, 2009b; Miyazaki, 2004a]. For rapidness, some musicians with a high level of AP could recognize the pitch of a given tone and press its corresponding key on a digital piano within 0.6 seconds on average [Miyazaki, 1990]. Another feature is that AP does not require explicit cognitive efforts, thus, those with AP spontaneously and unintentionally recognize the pitch of non‐musical ambient sounds such as the clicking sound of glasses [Miyazaki, 2004a]. The level of automaticity of AP is very high, hence, it is usually difficult to control (although it is possible through training) as demonstrated by Stroop‐like effects, namely slower and inaccurate responses to incongruently labeled tones [Akiva‐Kabiri and Henik, 2012; Itoh et al., 2005; Schulze et al., 2013]. Apart from in musical composition and conducting, AP is not generally regarded as being musically beneficial. Instead, it could even be disadvantageous for players or singers because of the uncontrollability. For instance, musicians with AP may have difficulties recognizing transposed melodies [Miyazaki, 2004b].

### Previously Found Neural Correlates of AP

From a cognitive perspective, AP perception may be associated with two different processing steps: (1) an early perceptual categorization of pitch and (2) a late cognitive pitch labeling that involves verbal/motor association with the pitch representation [Levitin and Rogers, 2005]. As hinted by the rapid reaction time shown in behavioral experiments [Miyazaki, 1990], both processing steps appear to be very fast. In an electroencephalography (EEG) experiment [Itoh et al., 2005], an early event‐related potential (ERP) component called “AP negativity” (with a latency of about 150 ms from the left posterior temporal electrode) was observed from AP musicians regardless of tasks (i.e., either passive listening, pitch‐naming, or Stroop tasks) compared to participants without AP. The Stroop‐like effect (i.e., incongruent labeling of pitch) modulated the amplitude of the AP negativity, which was interpreted as evidence of an automatic process that associates pitch and pitch label [Itoh et al., 2005].

Throughout neuroimaging morphometric literature using magnetic resonance imaging (MRI), a leftward asymmetry of the planum temporale (PT) has been identified as a key anatomical feature of AP. In early studies based on manual delineation of the region‐of‐interest (ROI) [Keenan et al., 2001; Schlaug et al., 1995], the area of the right PT was found to be smaller in AP musicians compared to non‐AP musicians, which increased the leftward asymmetry index of the PT area in musicians with AP. A voxel‐based morphometry (VBM) study [Luders et al., 2004] also reported leftward asymmetry in local gray matter volume (GMV) in PT from a larger sample, although the authors did not report whether the result was due to a smaller GMV in the right PT or a larger GMV in the left PT [Luders et al., 2004]. A more recent VBM study again demonstrated a smaller volume of the right PT in AP musicians [Wilson et al., 2009]. These findings suggest that AP ability is associated with a smaller PT in the right hemisphere.

On the functional side, greater activation in AP as compared to non‐AP musicians has been consistently found in the left posterior supra‐temporal plane (STP) including PT from electrophysiological data [Itoh et al., 2005], functional MRI (fMRI) [Ohnishi et al., 2001; Schulze et al., 2013], and positron emission tomography (PET) evidence [Wilson et al., 2009]. Moreover, greater activation in the left frontal cortex was found during passive listening [Ohnishi et al., 2001; Wengenroth et al., 2014], processing incongruently labeled tones [Schulze et al., 2013], and pitch naming [Wilson et al., 2009; Zatorre et al., 1998]. From these functional studies it appears that AP‐related processing does occur in the left hemisphere, possibly in relation to language processes such as verbal labeling.

Conversely, Wengenroth and colleagues reported greater volume of the right Heschl's gyrus (HG) in AP as compared to non‐AP musicians [Wengenroth et al., 2014]. In that study, the posterior border of the right HG is extended posteriorly, possibly explaining the smaller area (or volume) of the right PT in previous morphometric studies [Keenan et al., 2001; Schlaug et al., 1995; Wilson et al., 2009]. Their finding of a larger right HG was accompanied by a larger amplitude of a dipole model fitted to the P2a component of the auditory evoked potential, where the dipole was located in the anterior PT of the right hemisphere [Wengenroth et al., 2014]. Moreover, BOLD activation during passive listening was correlated with the behavioral score of AP in the right PT, but not in the left PT [Wengenroth et al., 2014].

To summarize, so far there is evidence of an increased leftward asymmetry for the posterior STP (i.e., PT) and an increased rightward asymmetry for the HG, in terms of gray matter volume and activation. The hemispheric specialization of AP processing requires further investigation, possibly by employing different methods which are more sensitive to the fine structure of the neural tissue.

### Towards a Computational Model of AP

The neuroimaging studies mentioned above indicate important candidates for the neural substrate of AP by describing macroscopic morphology and activations. However, to understand how AP perception is implemented in the neural circuitry, detailed structural information is essential. Recently, in vivo imaging of myeloarchitecture of the human cortex has drawn much attention in the neuroimaging field [Blackmon et al., 2011; De Martino et al., 2014; Dick et al., 2012; Glasser and Van Essen, 2011; Hashim et al., 2015; Lutti et al., 2014; Shafee et al., 2015; Sigalovsky et al., 2006] due to its significant implication in the working principles of the human cerebral cortex [Nieuwenhuys, 2013]. Unlike conventional morphometric methods that only use arbitrary image intensity of T1‐weighted images, such as VBM [Ashburner and Friston, 2000] or cortical thickness analysis [Chung et al., 2005], this novel technique can infer the myelin content in a voxel based on the inverse relationship between myelin content and quantitative longitudinal relaxation time (qT1) [Marques et al., 2010]. Experiments that demonstrated the relevance of intracortical myelin to brain function and behavior already exist [Grydeland et al., 2013, 2015]. Moreover, applications of in vivo myelin mapping demonstrated that the separation of the core and belt regions of the human auditory cortex is feasible by in vivo imaging [De Martino et al., 2014; Dick et al., 2012] based on the same myeloarchitectonic principles that are used in histological studies, namely, the core is defined by denser myelination [Wallace et al., 2002].

Intracortical myelination may be beneficial for neural circuits that enable AP, because cortical myelin provides electric insulation that reduces ephaptic crosstalk between nearby axons and thereby contributes to the specificity of transmission. That is, cortical myelin may support higher precision in categorization of pitch chroma. Moreover, cortical myelination development is known to suppress neuroplasticity after the “critical period” [McGee et al., 2005]. This fixation effect of cortical myelination is particularly interesting given the known importance of musical experience during the early stage of life (i.e., between the ages of 4 and 7 years) in acquiring AP [Baharloo et al., 1998; Miyazaki, 2004a, 2012]. Thus, greater cortical myelination may indicate that more information is preserved in neural circuits, which lasts throughout life.

Hence, the degree of axonal myelination could be an important structural property of neural tissue with respect to AP ability, which goes beyond the mere size (area or volume) of macroscopically delineated sections of the cortex. This hypothesis will be tested in the present study by comparing cortical myelination between musicians with and without AP.

If the above hypothesis is confirmed and myelination is discriminative between AP and non‐AP brains, we will also be able to shed new light on the location of the AP processor. Importantly, such a result is expected to directly point to the locations where increased or altered computational procedures associated to AP processing are carried out. Because of the speed and accuracy of AP processing indicated by behavioral investigations [Miyazaki, 1990] and the short latency of the AP‐specific ERP component [Itoh et al., 2005], it is highly likely that early auditory processing is involved in chromatic categorization. Thus it is reasonable to expect greater myelination in regions of the auditory cortex (either primary or non‐primary).

Finally, in light of cortical myelination, we will elucidate the question whether chromatic processing in AP musicians is spatially distinct from mere frequency discrimination, as suggested by previous behavioral evidence. In an early study [Siegel, 1974], AP listeners showed better pitch discrimination performance than control listeners only when the reference tone was tuned in to standard pitch (i.e., A4 = 440 Hz). This advantage of AP musicians was abolished when the tones were deviantly tuned (i.e., A4 is not exactly 440 Hz), implicating that pitch discrimination ability is independent of AP acuity, as extensively discussed in the literature [Deutsch and Henthorn, 2004; Miyazaki, 1988, 2004a; Takeuchi and Hulse, 1993]. Accordingly, we hypothesize that myeloarchitectonic features related to the frequency discrimination threshold (FDT) [Micheyl et al., 2006] would be found in auditory cortical regions that are spatially distinct from the myeloarchitectonic correlates of AP. More specifically, because pitch‐selective neurons were found near the anterolateral border of primary auditory cortex in marmoset monkeys [Bendor and Wang, 2005] and at the anterolateral end of HG in humans [Penagos et al., 2004], we hypothesize that pitch discrimination precision may be related to myeloarchitecture in the lateral HG extending to superior temporal gyrus (STG).

To achieve these aims, we map quantitative longitudinal relaxation rates (qR1 = 1/qT1) in the cortex using ultra‐high‐field (7T) MRI, which allows for submillimeter resolution, and we investigate whether and where there is a relationship between the concentration of myelin in the cortex on the one hand, and the AP and FDT abilities on the other**.**

## MATERIALS AND METHODS

### Participants

Eight AP musicians (five women) and nine non‐AP musicians (five women) participated in MRI and behavioral experiments at the Max Planck Institute for Human Cognitive and Brain Sciences in Leipzig, Germany. Participants were recruited via the Institute's participant database and flyers posted in the University of Music and Theatre “Felix Mendelssohn Bartholdy” Leipzig and the University of Leipzig. Inclusion conditions comprised: age between 18 and 40 years, musical training of more than 10 years, right‐handedness (i.e., laterality coefficients of handedness [LQ] ≥ 70 [Oldfield, 1971]), absence of any contraindication for MRI scanning, and absence of any history of neurological disorders, psychiatric diseases, use of psychiatric medications, head trauma, hearing loss, and tinnitus. An abbreviated audiometry, which was used to equalize stimuli presentation loudness across participants, confirmed intact hearing of all musicians. Prior to recruitment of musicians who identified themselves to have AP, a web‐based AP test was used to confirm it (≥80% correct answers). There was one case of self‐reported absence of AP, which was re‐categorized into the AP group based on that test. The local ethics committee approved the experimental protocol, and all participants submitted written informed consents prior to experiments.

Demographic information of AP and non‐AP musicians is listed in Table 1. The musical aptitudes were estimated by the “Musical Ear Test” [Wallentin et al., 2010]. Here, only the scores of the melodic tasks are reported, while rhythmic tasks are left out. Frequency discrimination threshold (FDT = F1/F0; F1 and F0 are the frequencies of target and reference, respectively) was measured using an abbreviated version of a protocol presented in Micheyl et al. [2006]. Sex, age, handedness, musical aptitude, and logarithmic FDT were matched between the groups (minimum *P =* 0.107). The first musical instrument was for the AP musicians (*n =* 9): piano (*n =* 6), violin (*n =* 1), guitar (*n =* 1), and clarinet (*n =* 1); for the non‐AP musicians (*n =* 8): piano (*n =* 5), violin (*n =* 2), and guitar (*n =* 1).

**Table 1:** Mean and standard deviation of demographic information

	Non‐AP (n = 9)		AP (n = 8)			
Variables	Mean	Std.	Mean	Std.	*t*/*z*‐statistic	*P*
Sex ratio (women/all)	0.56	—	0.62	—	0.22	0.823
Age (yr)	25.78	5.19	26.88	2.95	0.53	0.607
Handedness (LQ)	90.56	11.54	92.63	10.60	0.87	0.399
MET‐melody hit rate (%)	82.26	11.12	91.11	10.02	1.71	0.107
FDT^a^	2.41	0.23	2.73	0.68	−1.34	0.199
Ethnicity ratio (Asian/all)	0.00	—	0.38	—	2.91	0.004
Musical training onset (yr)	8.00	2.96	5.00	1.51	−2.88	0.012
Musical training duration (yr)	16.9	7.83	23.00	3.34	2.04	0.059

a FDT is transformed in negative logarithm base to 10.

LQ, laterality coefficient, from −100 (exclusively left‐handed) to 100 (exclusively right‐handed) [Oldfield, 1971]; MET, Musical Ear Test [Wallentin et al., 2010]; FDT, frequency discrimination threshold [Micheyl et al., 2006]; Std., standard deviation.

There were mismatches between the AP and non‐AP groups in terms of ethnicity. This imbalance is rooted in the much higher incidence of AP in Asian as compared to European musicians [Miyazaki et al., 2012], which makes it difficult to recruit balanced cohorts. Nonetheless, using a simulation, we demonstrated that the reliability in estimating the effect size of AP under the current design (relative error = −0.09 ± 13.16%) is still comparable to a perfectly balanced design (relative error= 0.05 ± 9.41%) and much better than the worst case (relative error = −0.39 ± 21.39%). See Supporting Information for further details about this simulation. In an additional analysis, only with European participants (see Results below), we replicated the main finding even from that small subset, which means that the result cannot be due to confounding influence from ethnicity and therefore this speaks for the robustness of the findings.

### Behavioral Tests

The AP performance of all musicians was measured by a behavioral test where the participants heard a random sequence of pure and piano tones tuned to the 12‐key equal‐tempered scale with A4 as 440.0 Hz and then pressed the corresponding keys on a muted digital piano. The target tones spanned 3 octaves: C3 (130.81 Hz) to B5 (987.77 Hz) for sine waves and E3 (164.81 Hz) to D#6 (1,244.50 Hz) for piano timbre. The pure tones had a length of 1 s and were prepared with the sine function of MATLAB (version 8.2; Mathworks Inc., Natick, MA) with linear ramps of 10 ms at the beginning and the end. The piano tones were also 1 s long, created using music creation software called GarageBand (version 10.1.0; Apple Inc., Cupertino, CA) with a virtual musical instrument named “Steinway Grand Piano.” Monochannel audio files were sampled at 44,100 Hz with a precision of 16 bits per sample. Each target tone was presented once, resulting in 72 trials (36 pure + 36 piano tones). Stimuli were binaurally delivered through headphones (HS‐800; A4tech, Taipei, Taiwan) at the approximate intensity of 70 dB sound pressure level (SPL) to a participant sitting in front of an 88‐key digital piano (CLP‐150; Yamaha, Hamamatsu, Shizuoka, Japan), which was linked to the experiment software package Presentation (version 14.9 build 20110719; Neurobehavioral Systems Inc., Berkeley, CA) on the Windows XP system (version 5.1.2600; Microsoft, Redmond, WA) via musical instrument digital interface (MIDI).

To differentiate interindividual variability in AP performance, we introduced a limited response time window from the stimulus onset to 4 s after the onset. Participants were instructed to respond as quickly as possible while maintaining accuracy within the given time window of 4 seconds. They were warned about time‐out after 4 s. A short training session was used to familiarize the participants with the experimental procedure.

The absolute pitch performance was analyzed in terms of (1) absolute error (AE), (2) absolute octave‐error‐corrected error (ACE), (3) difference between mean AE and mean ACE, as a measure of octave error, (4) AP score (APS), (5) hit rate, and (6) reaction time (RT) for pure and piano tones. “Octave‐error” is defined by an answer with correct pitch chroma but incorrect pitch height, which is known to be frequently observable in AP musicians [Miyazaki, 1988, 2004a]. Octave error correction was achieved by disregarding errors with respect to octaves: for example, if the target is “C4” and the participant's answer is “C3,” the error would be 12 semitones for AE, but 0 semitones for ACE. Arithmetically, the pitch chroma in the 12‐tone key scale can be noted by a remainder after division of a natural index of a pitch in the unit of semitone (e.g., MIDI codes) by 12 as c∈{0, 1, 2,⋯, 11}. An octave‐error corrected error *E* between two pitch chromas *c*_1_ and *c*_2_ (*c*_1_ > *c*_2_) is given as:

(1) $$E=\{\begin{array}{cc} c_{1}-c_{2} & \text{if}\;\;c_{1}-c_{2}\leq 6\\ -\{c_{2}-(c_{1}-12)\} & \text{if}\;\;c_{1}-c_{2}>6. \end{array}$$

A positive, scaled score of AP (APS) was computed from ACE as ${\text{APS}}_{j}=1-m_{j}/m_{\max}$ for the *j*th timbre, where *m* is the mean ACE, and $m_{\max}$ is the maximum of possible corrected errors, which is 6 semitones. Thus the APS is confined between 0 and 1, positively coding the AP ability with the chance level of 0.5.

### Image Acquisition and Processing

Magnetization‐prepared two rapid gradient echo (MP2RAGE) [Marques et al., 2010] images at 0.7 mm isotropic resolution were acquired using a 7‐T whole‐body MR system (Siemens, Erlangen, Germany) with an eight‐channel head coil system (RAPID MR International, OH). From the two inversion images, a T1‐weighted (T1w) image and a qT1 image were derived [Marques and Gruetter, 2013]. The imaging parameters were: TR/TE/TI1/TI2 = 5,000/2.45/900/2,750 ms, FA1/FA2 = 5/3 degrees, FOV= 223 × 223 mm^2^, image matrix = 320 × 320 × 240 (TR, time of repetition; TE, time of echo, T1, time of the first inversion; T2, time of the second inversion; FA, flip angle; FOV, field of view).

The qT1 images were mapped onto cortical surfaces generated by FreeSurfer [Fischl, 2012] at various depths (i.e., 25, 50, and 75% of cortical thicknesses, measured from the interface between gray and white matter). Subsequently, the qR1 (= 1/qT1) values were computed from the surface‐mapped qT1 values and bounded within a range between 0.25 and 10 s^−1^, which corresponds to the qT1 range between 100 and 4,000 ms. Following this, they were registered onto the template meshes. A detailed description of the image processing can be found in the Supporting Information.

### Statistical Inference

Statistical tests were carried out using a general linear model (GLM) for each layer and each hemisphere with the nuisance covariates age, sex, and ethnicity. Multiple comparison correction based on the Random Field Theory (RTF) was applied after surface‐based smoothing with a two‐dimensional Gaussian kernel with the full‐width of half‐maximum (FWHM) of approximately 8 mm (10 iterations on the mesh with a mean edge length of 0.77 mm) using the SurfStat Toolbox [Worsley et al., 2009] on MATLAB environment. Due to signal dropout in the inferior regions of the brain in the original 7 T measurements, we limited the search region to accurately estimate the number of resels, or resolution element in RTF [Worsley et al., 1992] by excluding ventral regions (i.e., temporal poles, fusiform gyri, entorhinal cortices, parahippocampal gyri, and inferior temporal lobes) as well as the medial wall (a cut section of the corpus callosum and subcortical structures to separate hemispheres) using an automatic parcellation based on the “Desikan‐Killiany‐Atlas” [Desikan et al., 2006] from the FreeSurfer package. Because the signal drop is dependent on the position in the head coil, systematic bias due to gross morphology such as the inferior‐to‐superior length of a head could alter qR1 values and statistical inference unless masked. In the current study, the family‐wise error rate (FWER) was controlled to be below 0.05 at the cluster level with a cluster‐defining threshold of 0.001.

## RESULTS

### Behavioral Results

In our experiment we allowed responses only within 4 s after the stimulus onset, causing some of the trials to be left unresponded. Additionally, in some cases, multiple keys were pressed within the time window. If the first response was given later than 100 ms after the stimulus onset, it was taken as a valid response; otherwise it was regarded as a late response to the previous trial. The overall percentage of valid responses was 93.7 ± 8.1% (non‐AP) and 94.4 ± 1.0% (AP).

**Figure 1:**
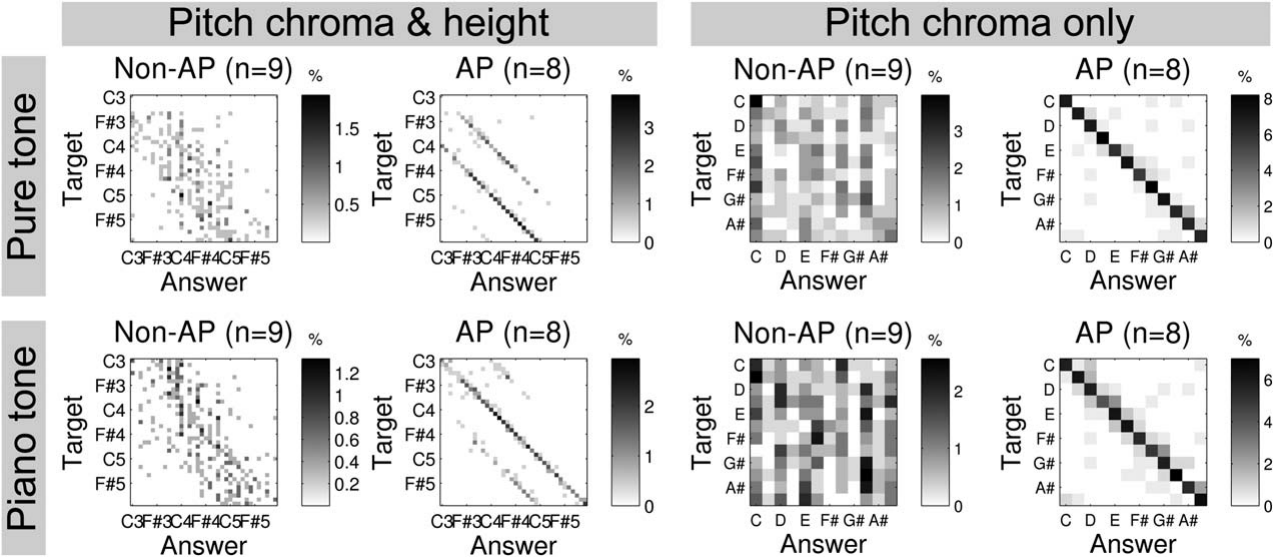
Confusion matrices from the absolute pitch (AP) test for non‐AP and AP groups. For each matrix, rows correspond to the target notes and columns correspond to the answers of the participants. For pure tones (upper row) and piano tones (lower row), the four matrices on the left side show raw responses (“Pitch chroma & height”) and octave error corrected responses (“Pitch chroma only”). Gray scale codes the proportion of the trial numbers for a specific combination of target and answer notes over the total number of trials.

The group differences in the AP test are illustrated using confusion matrices in Figure 1. The row of each matrix corresponds to the pitch of a presented tone. The column corresponds to the pitch of the answer: An element at the *i*th row (e.g. C4) and *j*th column (e.g., C3) represents how often (on average) subjects reported the *j*th note (e.g., C3) when the target was actually the *i*th note (e.g., C4). AP musicians showed distinctively sharp diagonal elements (meaning correct answers) whereas non‐AP musicians did not show such a pattern, but a very broad scatter over nearly one octave. As commonly reported in the literature [Miyazaki, 1988; Takeuchi and Hulse, 1993], it was notable that AP musicians also made “octave errors,” which are defined as answers with correct pitch chroma but incorrect pitch height. In accordance with a previous study [Miyazaki, 1989], AP musicians made more octave errors with pure tones (59.6 ± 19.3% of total valid answers) than with piano tones (14.6 ± 12.3% of total valid answers; *P <* 0.0001), which could be explained by the relative unfamiliarity of the timbre of sine waves to musicians. There was no correlation between the frequency of octave errors and the APS (see Supporting Information).

Table 2 summarizes the statistics of AP performance including “hit rates” (regarding errors up to one semitone as ‘hit’) **i**n order to enable comparisons to previous studies [Keenan et al., 2001; Schulze et al., 2013]. The mean values of AE were smaller in AP only for piano tones (*P =* 0.028) but not for pure tones (*P =* 0.777). In contrast, the mean values of ACE were smaller in AP both for pure and piano tones (*P <* 10^−6^). The indifference in AE for pure tones is possibly due to the persistent octave errors in some musicians with AP (e.g., in an extreme case, octave errors were found in 83% of valid responses). The hit rates were also significantly different between APs and non‐APs (*P <* 10^−5^), consistently with the literature [Keenan et al., 2001; Schulze et al., 2013]. The average hit rates were slightly lower than in previous studies [Keenan et al., 2001; Schulze et al., 2013] because those studies applied a more stringent threshold (>90%) [Miyazaki, 1988]. In our case, hit rates from two musicians were below 90%, thus those participants could be classified as “quasi‐APs” [Wilson et al., 2009]. Because of this, we also did correlation analysis with APS, which was measured for all musicians.

**Table 2:** Behavioral measures for absolute pitch performance

		Non‐AP (*n* = 9)		AP (*n* = 8)			
Measure	Timbre	Mean	Std.	Mean	Std. dis	*t*‐statistic	*P*
AE (semitones)	Pure tone	11.44	6.90	10.81	4.52	0.22	0.831
	**Piano tone**	**8.59**	**5.05**	**3.69**	**2.14**	**2.54**	**0.023**
ACE (semitones)	**Pure tone**	**2.89**	**0.26**	**0.36**	**0.51**	**13.18**	**<10^−8^**
	**Piano tone**	**2.99**	**0.43**	**0.53**	**0.57**	**10.11**	**<10^−6^**
APS	Pure tone	0.52	0.04	0.94	0.08	−13.18	<10^−8^
	Piano tone	0.50	0.07	0.91	0.10	−10.12	<10^−7^
Hit rate^a^	**Pure tone**	**0.27**	**0.07**	**0.87**	**0.21**	**8.31**	**<10^−5^**
	**Piano tone**	**0.29**	**0.09**	**0.89**	**0.17**	**9.02**	**<10^−5^**
RT (s)	Pure tone	2.02	0.64	1.59	0.65	1.36	0.194
	Piano tone	1.92	0.48	1.69	0.74	0.79	0.441

Significantly different measures are marked with a bold type (*P <* 0.05).

a An error with a semitone was considered as a correct response as in some of previous literature [Keenan et al., 2001; Micheyl et al., 2006; Oldfield, 1971; Schulze et al., 2013].

AE, absolute error; ACE, absolute corrected error; APS, absolute pitch score; RT, reaction time; Std., standard deviation.

Unexpectedly, no significant difference in the RT was found between non‐AP and AP musicians (min *P =* 0.179), although the participants were instructed to respond as quickly as possible while maintaining the accuracy of responses within the 4‐s time window. The mean RTs in the current study (1.97 s in non‐AP; 1.64 s in AP) were much smaller than in a previous study that allowed self‐paced timing (7.6 s in non‐AP; 3.3 s in AP) [Bermudez and Zatorre, 2009a]. Because of the fixed response window, the non‐AP participants could not take longer than AP participants as in the previous study [Bermudez and Zatorre, 2009a], which presumably is the reason for the observed lack of difference in RTs.

### Intracortical Myelination and Absolute Pitch

**Figure 2:**
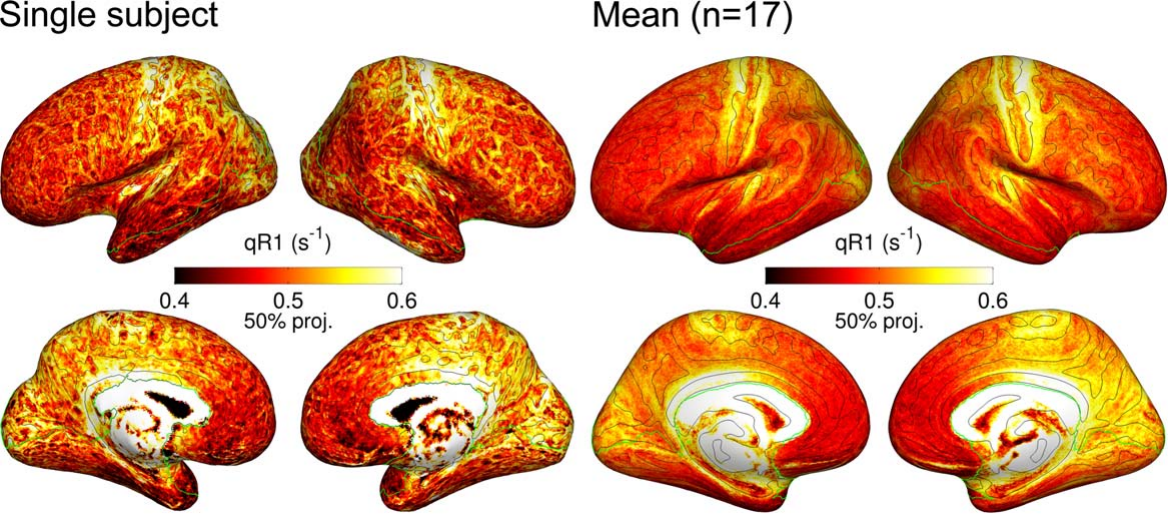
Surface‐mapped quantitative longitudinal relaxation rate (qR1) values of a single subject (left) and the average over all subjects (right, *n =* 17). qR1 values were sampled at the 50% projection level of cortical thickness from the white matter surface. Masks excluding ventral regions are indicated by green contours (see Methods, Statistical Inference). Note that the values of the single subject were minimally smoothed for visualization (FWHM = 1 mm) whereas the group mean values were not smoothed. Isocurvature contours (black) at zero level are overlaid to aid localization of landmarks of the inflated cortical surfaces. [Color figure can be viewed in the online issue, which is available at http://wileyonlinelibrary.com.]

Examples of surface‐mapped qR1 values of a single subject and the total mean (*n* = 17) are shown in Figure 2. In high accordance with previous in vivo myelination studies [De Martino et al., 2014; Glasser and Van Essen, 2011; Shafee et al., 2015], high qR1 values (indicating high myelination) were observed in primary motor/somatosensory cortices (the pre/postcentral gyri), primary auditory cortex (the medial region of Heschl's gyrus), and primary visual cortex (the occipital poles and the precuneus).

In order to find myeloarchitectonic correlates of the AP ability, it is desirable to control for confounding factors. Age [Good et al., 2001b; Thambisetty et al., 2010], sex [Good et al., 2001a; Gur et al., 1999], and ethnicity [Chee et al., 2011] are known to correlate with macroscopic morphology such as local gray matter volume and cortical thickness. Moreover, age effects of cortical myelination have been reported [Grydeland et al. 2013; Shafee et al. 2015]. In particular, as the demographic variables significantly affected qR1 values in our current dataset (see Supporting Information for effects of demographic variables), we tested the effect of AP while controlling for possible confounding effects of these variables using a GLM as:

(2) $${\text{qR}}_{1}=\beta_{0}+\text{age}\cdot \beta_{1}+\text{sex}\cdot \beta_{2}+\text{ethnicity}\cdot \beta_{3}+\text{AP}\cdot \beta_{4}+\varepsilon ,$$

where qR_1_ is a column vector with one value per vertex; *age, sex, ethnicity, and AP* are column vectors coding age (years), sex (male or female), ethnicity (Asian or European) of musicians, and AP level (non‐AP or AP); $\beta_{i}$ is the *i*th unknown coefficient to estimate; ε is zero‐mean Gaussian noise.

**Figure 3:**
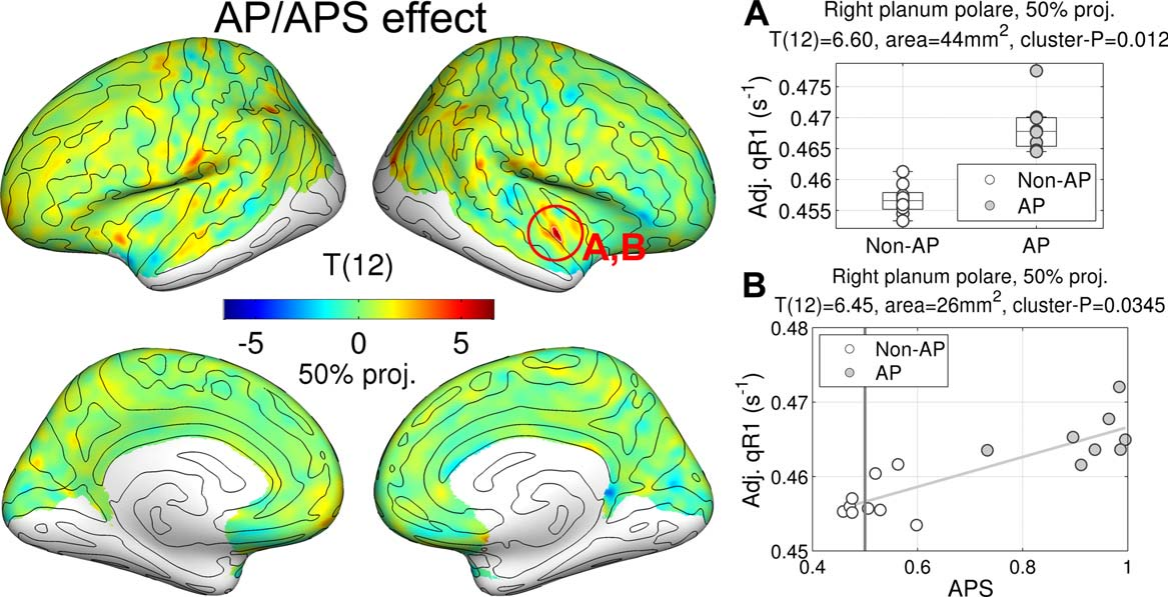
Effects of absolute pitch (AP) and AP score (APS) in quantitative longitudinal relaxation rate (qR1). *T*‐statistic maps for group differences are shown on inflated cortical surfaces. T‐statistic maps for APS effect are not shown because they are very similar to those of AP effect. The degree of freedom is noted in parentheses above the color bar. Regression plots for AP effect (**A**) and APS effect (**B**) are given. The expected APS by chance (0.5) is marked by a gray vertical line in the plot (B). Note that the qR1 values in scatterplots are adjusted for the nuisance variables in the GLM [Eq. (2)]. [Color figure can be viewed in the online issue, which is available at http://wileyonlinelibrary.com.]

We found greater qR1 values in the AP group compared to the non‐AP group in the right planum polare (PP) (max *T*_(12)_ = 6.60, *P* = 0.012, area = 40 mm^2^ at 50% projection level, peak MNI305‐coordinate = [46, −8, −12] mm) as shown in Figure 3A.

Furthermore, we analyzed the correlation between qR1 values and APS. Because APSs for pure tones and piano tones were very similar (*r* = 0.96, *P <* 10^−8^), the APSs for the two timbres were averaged for the correlational analysis. We used the same GLM [Eq. (2)] replacing the binary variable of AP group membership with a continuous behavioral variable of APS. We found a significant positive correlation in the right PP in the same position as for the AP (max *T*_(12)_ = 6.45, *P* = 0.035, area = 23 mm^2^ at 50% projection level, peak MNI305‐coordinate = [46, −5, −12] mm) as shown in Figure 3B.

**Figure 4:**
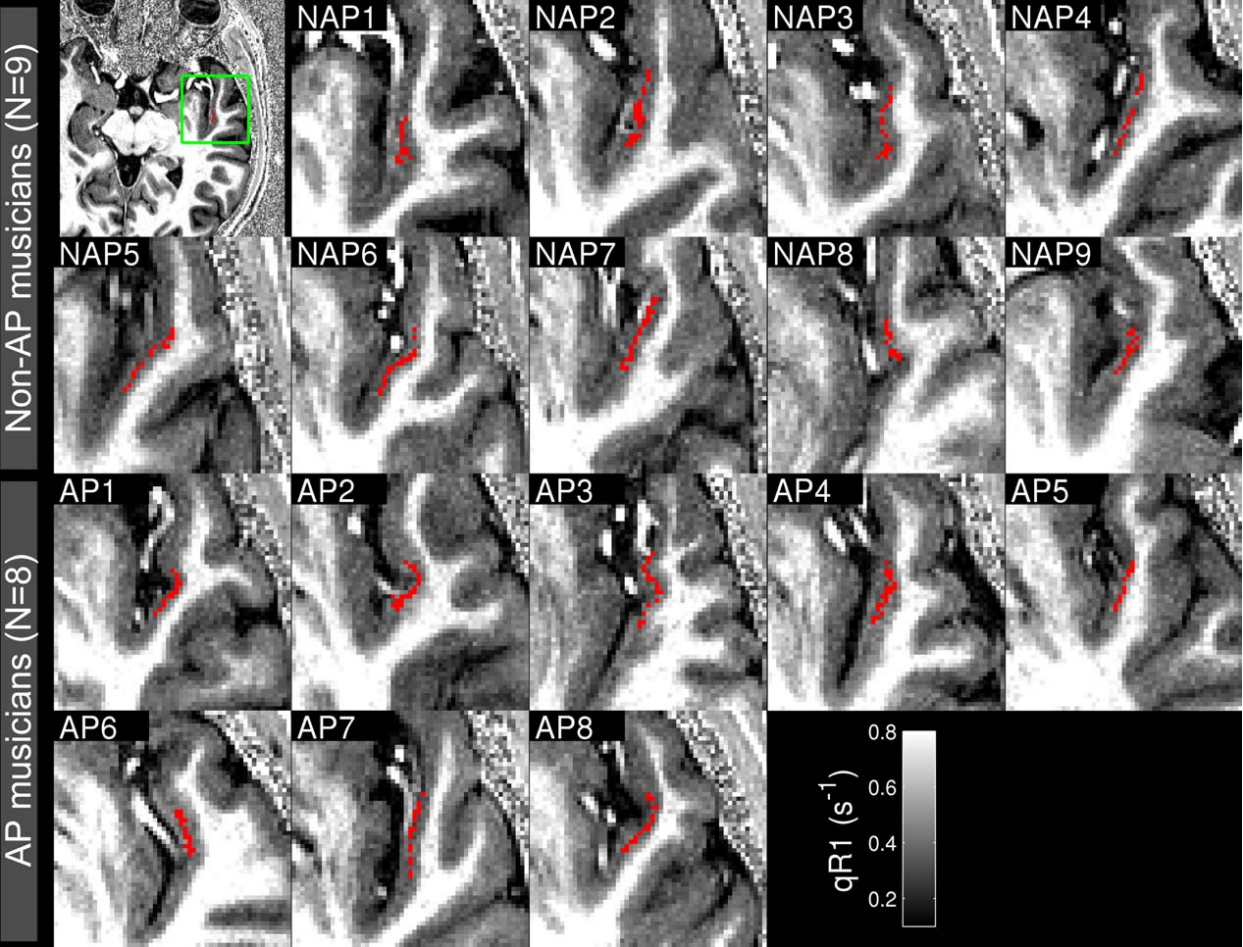
Axial slices showing significant cluster voxels (red) in native volumes of individuals. NAP, nonabsolute pitch musician; AP, absolute pitch musician; qR1, quantitative longitudinal relaxation rate. [Color figure can be viewed in the online issue, which is available at http://wileyonlinelibrary.com.]

In order to show anatomical landmarks around the significant clusters with AP‐related higher myelination, the significant clusters for the AP contrast in Eq. (2) were transformed back into the native volumes of participants as shown in Figure 4. The cluster voxels are aligned along the medial part of the PP in the right hemisphere.

### Ruling Out Confounds From Ethnicity: Effect of AP With Europeans Only

Although ethnicity was included in the previous analysis as a nuisance variable, one may still be concerned about the risk of confound. In order to demonstrate that the AP effect in the right PP cannot be due to the mismatch in ethnicity, we replicated the same analysis with only the European musicians (i.e., three AP Europeans vs. nine non‐AP Europeans). Because there was no Asian musician included in this subset, the ethnicity term was discarded as:

(3) $${\text{qR}}_{1}=\beta_{0}+\text{age}\cdot \beta_{1}+\text{sex}\cdot \beta_{2}+AP\cdot \beta_{3}+\varepsilon .$$

**Figure 5:**
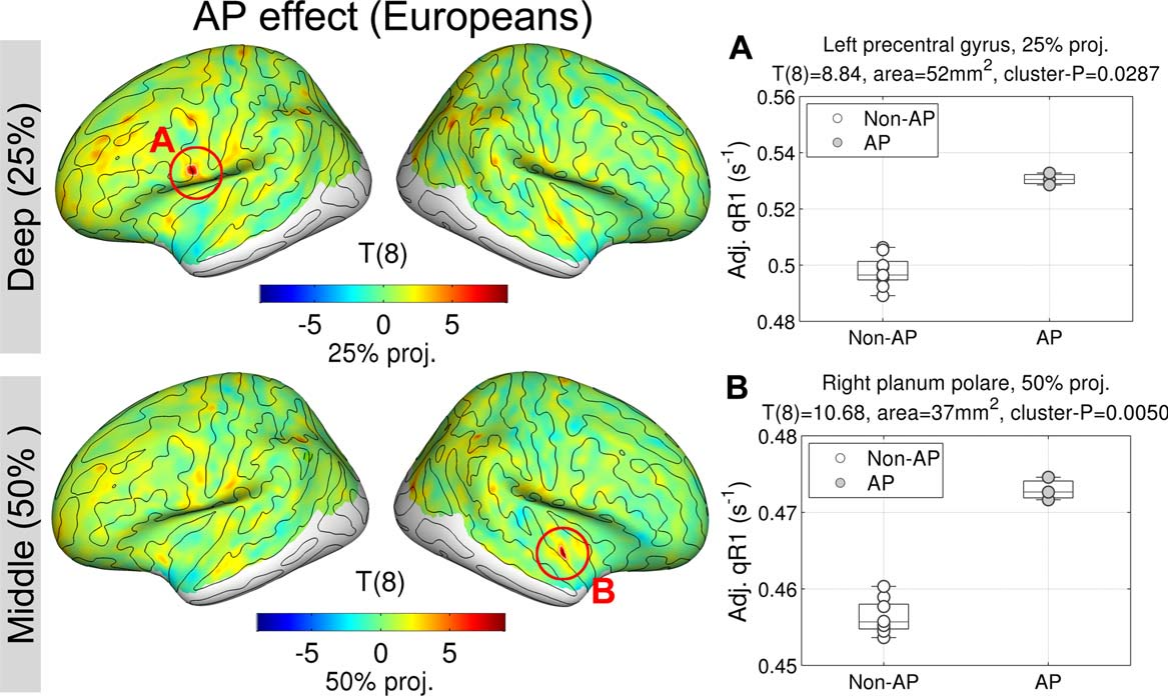
Effect of absolute pitch (AP) within only European musicians in quantitative longitudinal relaxation rate (qR1). *T*‐statistic maps are shown at the deep (25% of thickness) and the middle (50% of thickness) projection levels from the white matter surfaces. Medial views are omitted because no significant differences were found there. Note that the qR1 values in scatterplots are adjusted for nuisance variables. [Color figure can be viewed in the online issue, which is available at http://wileyonlinelibrary.com.]

Please note that, due to the small number of samples in one group (i.e., three APs), the sensitivity of the analysis is reduced. However, if it does yield a significant result, it cannot be confounded by ethnicity. The result is shown in Figure 5. At the same level in the same location, we reproduced the AP effect (max *T*_(8)_ = 10.68, *P* = 0.005, area= 37 mm^2^ at the 50% projection level, peak MNI305‐coordinate = [45, −9, −11] mm). The effect size of AP at the peak in the right PP was 0.0171 s^−1^, which is very close to the effect size of AP estimated with the whole dataset (0.0169 s^−1^; relative error = 1.18%). If the current finding of greater myelin in the right PP had been due to confounding effect from the mismatch of ethnicity in experiment design, there should not be such a cluster without the Asian musicians. Therefore we can rule out the possibility of an ethnicity confound in the results presented in Figure 3. See Supporting Information for a simulation demonstrating the reliability of the estimation under the current design.

An additional cluster in the left precentral gyrus was found (max *T*_(8)_ = 8.84, *P =* 0.029, area = 52 mm^2^ at the 25% projection level, peak MNI305‐coordinate = [−56, 6, 15] mm). However, the estimated effect size (0.0344 s^−1^) was much larger than that of the whole dataset (0.0118 s^−1^; relative error = 192.3%). Thus, the AP effect in the left precentral gyrus might be because of certain characteristics that are specific to the three European AP musicians, which were cancelled out by the Asian musicians when the GLM [Eq. (2)] was fitted to the full dataset. Hence, any further interpretation of this finding based on the current data is difficult. A dedicated study explicitly targeting the ethnicity effect would be needed here.

### Intracortical Myelination and Frequency Discrimination Threshold

**Figure 6:**
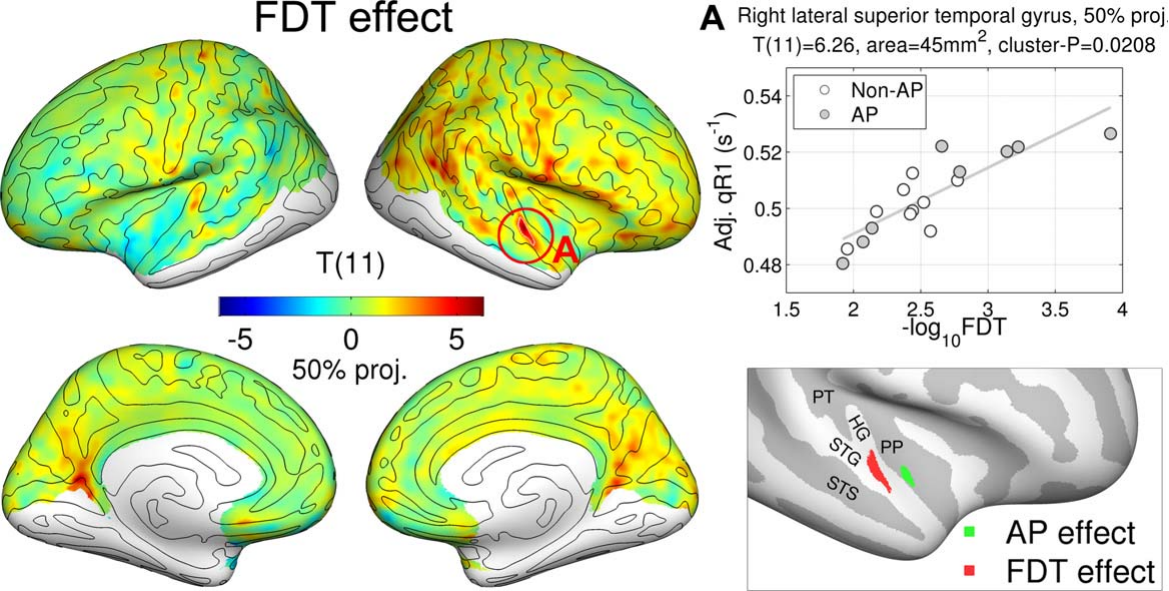
Effect of frequency discrimination threshold (FDT) in quantitative longitudinal relaxation rate (qR1). *T*‐statistic map only at the middle level (50% of thickness) is shown. A regression plot at the peak vertex is given with an inset showing locations of the significant clusters (*P* < 0.05) for AP effect (green) and FDT effect (red). For FDT effect, the cluster is a union of all clusters across layers. [Color figure can be viewed in the online issue, which is available at http://wileyonlinelibrary.com.]

Finally, to differentiate the observed neuronal correlate of AP from the sensitivity to mere pitch discrimination power (i.e., the relative pitch ability), we tested a GLM as:

(4) $$qR_{1}=\beta_{0}+\text{age}\cdot \beta_{1}+\text{sex}\cdot \beta_{2}+\text{ethnicity}\cdot \beta_{3}+\text{AP}\cdot \beta_{4}+(-\log_{10}\text{FDT})\cdot \beta_{5}+\varepsilon ,$$

where FDT = F1/F0 is the frequency discrimination threshold (see Methods section). Note that FDT was not significantly different between AP and non‐AP groups (see above). Strikingly, a localized positive correlation of cortical myelin with FDT was found in the lateral HG, extending into lateral STG, in the right hemisphere (max *T*_(11)_ = 6.26, *P =* 0.021, area = 56 mm^2^ at the 50% projection level, peak MNI305‐coordinate = [60, −1, −2] mm) as shown in Figure 6. We also found other regions in addition to HG (i.e., inferior frontal gyrus and intraparietal sulcus) in the superficial layer (see Supporting Information for scatterplots of all clusters). It is noteworthy that the significant cluster of the AP group effect in the anteromedial part of STP (Figure 8, inset, green) did not overlap with the effect of FDT in the anterolateral part of STG (Figure 8, inset, red). This clearly demonstrates the distinctiveness of the AP‐related temporal myeloarchitecture (for pitch chroma recognition) and the FDT‐related one (for relative comparison of two successive tones).

## DISCUSSION

In the search for the myeloarchitectonic correlate of AP, we found greater myelination in AP musicians compared to non‐AP musicians in the right PP. This is, to our best knowledge, the first in vivo finding of myeloarchitecture that correlates with absolute recognition of pitch chroma. As hypothesized, the group effect of AP and the correlation effect of APS were located in the STP. In addition, we found a positive correlation with the frequency discrimination threshold in the lateral Heschl's gyrus demonstrating spatially distinct neural processes of absolute recognition of pitch chroma and relative frequency resolution.

While our findings associate the AP ability with the myeloarchitecture of the right anterior STP that is part of the ventral auditory pathway, some previous studies have found heightened leftward specialization in the posterior STP (i.e., the area, volume, GMV as well as the functional activation of the PT) in AP musicians compared to non‐AP musicians [Keenan et al., 2001; Luders et al., 2004; Schlaug et al., 1995]. In addition, a recent diffusion tensor imaging study reported greater fractional anisotropy (FA) in the left superior longitudinal fascicle (i.e., the dorsal auditory pathway) [Oechslin et al., 2009]. Although this might seem surprising at first glance, it is not necessarily a contradiction, because we analyzed a different aspect of anatomy, namely cortical myelination as opposed to cortical thickness [Dohn et al., 2015], area size [Keenan et al., 2001; Schlaug et al., 1995], or diffusion anisotropy in the white matter [Oechslin et al., 2009] in previous studies. These aspects do not need to be correlated. For example, an increase or decrease of GMV at the border of the cortex would affect the image intensity gradient, thus changing the apparent cortical thickness. However, in the present study, we found an increase in myelination in the middle depth of the cortex, which is less likely to affect the cortical surface reconstruction [Dale et al., 1999]. As previously discussed in the Introduction, the notion of leftward asymmetry of AP processing [Keenan et al., 2001; Schlaug et al., 1995] is not uncontended, because recent studies reported AP effects also in the right STP [Bermudez et al., 2009; Dohn et al., 2015; Wengenroth et al., 2014]. In particular, a positive correlation between the right HG volume and AP proficiency suggests that AP processing is not strictly left hemispheric (possibly bilaterally in parallel) [Wengenroth et al., 2014]. Moreover, because AP mainly involves processing spectral aspects of the auditory input, the idea of hemispheric differences in temporal integration time windows [Poeppel, 2003] and the hemispheric specialization theory [Zatorre and Zarate, 2012] support a prominent role of the right hemisphere. Here, we discuss the biological implications of intracortical myelination, particularly in the context of the role of the anterior STP in pitch chroma processing.

### Implication of the Intracortical Myelination for AP

Myelination in the cortex, and alterations thereof, can in principle be due to myelination of intracortical fibers or due to long‐range axonal connections that start from or end in the cortical region. In our study, myelination effects were found in the right STP at middle depth (i.e., 50% of cortical thickness) and not in deeper or more superficial cortical layers. Moreover, we did not find a group difference in qR1 values in the subcortical white matter underneath the right STP (see Supporting Information). Although the subcortical white matter needs to be further investigated using imaging techniques that are more sensitive to white matter microstructures, such as magnetization transfer imaging or diffusion‐weighted imaging, the absence of strong differences in the subcortical white matter, along with the restriction of the cortical findings to the middle layer, suggests that the myelination effect in the anterior STP is indeed mainly related to local connections.

Ephaptic crosstalk between two adjacent axons depends on the electric resistance between a node of Ranvier in one axon and the nearest, myelinated part of the other axon [Binczak et al., 2001]. By means of electrical insulation, myelination of local neural circuitries could reduce ephaptic coupling between nearby axons, therefore, increasing specificity of information transfer. Extraction of pitch chroma based on the long‐term templates of 12 tones in the Western scale would require precise processing of pitch information, which is already a cortical representation based on acoustic inputs [Griffiths and Hall, 2012]. If such neural operations occur in the non‐primary auditory cortex, greater local myelination might support processing with heightened specificity. It is known from postmortem investigations that nonprimary auditory cortex in the human brain has more widespread tangential intracortical connections compared to primary auditory cortex [Tardif and Clarke, 2001]. The current myeloarchitectonic findings might be interpreted as an alteration of the tangential connections in the nonprimary auditory cortex, which contribute to the computation of pitch chroma.

Moreover, intracortical myelination can restrict experience‐driven neuroplasticity. In a mouse model [McGee et al., 2005], an abrupt increase of gray matter myelination was found in normal mice after the critical period, which precluded further reorganization of ocular dominancy. The importance of cortical myelin in suppression of neuroplasticity in adult mice was also demonstrated by the observation that focal demyelination enabled recovery from amblyopia [Bavelier et al., 2010]. A recent intriguing human study found that administration of volproic acid, which is known to cause hypomyelination of the corpus callosum in developing mice [Shen et al., 2005], can enable human adults to acquire an AP‐like long‐term memory for a limited number of tones [Gervain et al., 2013]. Given that the acquisition of AP is associated with an early onset of musical training/experience, typically between age 4 and 7 years [Baharloo et al., 1998; Miyazaki and Ogawa, 2006], the greater myelination in the AP musicians may imply that AP requires a prohibitory mechanism of neuroplasticity, which preserves the spectro‐temporal templates obtained during the critical period in children with predispositions for AP [Baharloo et al., 2000].

### Functional Role of the Anterior STP in AP

We found heavier cortical myelination in the anterior STP in the AP musicians. Specifically, the myelin difference was localized on the medial part of the PP, which corresponds to anterior auditory (AA) region as defined based on cyto‐/receptor architecture [Rivier and Clarke, 1997]. The AA region, which is a part of the ‘belt’ region, was characterized by strong (but weaker than primary auditory area; AI) densities of soma and fibers of pyramidal cells in layer III [Rivier and Clarke, 1997]. A tracing study found that the intrinsic connectivity of area TG (in PP) is mainly of longer range (greater than 4 mm and up to 7 mm) compared to that of HG (less than 2.5 mm) and anisotropic: the connections spread more posteriorly towards HG [Tardif and Clarke, 2001]. Based on the intermediate cytoarchitectonic properties (i.e., cell density greater than lateral STG but smaller than AI) AA has been considered as an early non‐primary auditory cortex [Rivier and Clarke, 1997] and also a part of the ventral pathway based on neuroimaging studies that demonstrated selectivity of AA to sound recognition rather than sound localization [Clarke and Morosan, 2012].

The functional and structural dissociation of the spatial and non‐spatial information, being separately processed in parallel in non‐human primates [Kusmierek and Rauschecker, 2009; Rauschecker, 2015; Tian et al., 2001] and humans [Arnott et al., 2004; Warren and Griffiths, 2003], has been theorized as ‘dual‐auditory‐pathways’. From a number of human neuroimaging studies [Altmann et al., 2007; Arnott et al., 2004; Barrett and Hall, 2006; Hart et al., 2004], the spatial information was found to be processed in the dorsal auditory pathway whereas the nonspatial information was found to be in the ventral auditory pathway. Particularly, the ventral auditory pathway, including the right anterior region of STP and superior temporal sulcus (STS), has been believed to play a distinctive role in identifying auditory object properties [Griffiths and Warren, 2004], such as voice identity [Belin et al., 2000] and acoustic properties of vocal tracts [von Kriegstein et al., 2006]. This further demonstrated that the right anterior STS is sensitive to the change of the sound‐source identity using environmental sounds, of which distinctiveness was parametrically manipulated.

The differential processing of pitch chroma and pitch height has often been suggested based on the occurrence of octave errors in AP musicians [Deutsch, 2013; Deutsch and Henthorn, 2004; Miyazaki, 1988; Takeuchi and Hulse, 1993]. The dissociated recognition ability of AP musicians on pitch chroma and pitch height implies that the AP musicians are especially sensitive to the pitch chroma, not to the pitch height. A neuroimaging study that independently manipulated pitch chroma and pitch height revealed that the anterior STP is more sensitive to change in pitch chroma than the posterior STP [Warren and Griffiths, 2003], supporting the notion that recurring representation of pitch chroma is handled in a pathway that is distinct from the one that processes the pitch height. Thus, we speculate that the AP‐related myelination increase in the anterior STP found in our data might be related to the parallel processing of pitch chroma and pitch height. More specifically, for AP musicians, pitch chroma may be an identifiable property of an auditory object, while pitch height is processed as a relative position on a linear continuum, similarly to the non‐AP population. As discussed in the previous section, local myelination may increase specificity of the tone representations and hinder neuroplasticity [Bavelier et al., 2010], thus enabling precise matching between the spectrotemporal images [Griffiths and Warren, 2004] of online inputs and of the templates in long‐term memory.

Localization of the long‐term memory traces of pitch chroma in AP musicians remains unclear. Dohn et al. [2015] suggested an involvement of the right hippocampus based on their finding of a correlation between the FA of the white matter in the ventral pathway and cortical thickness in the parahippocampal gyrus. However, it was shown that the hippocampus is selectively involved in the retrieval of recollection‐like context‐based episodic memory, but not in familiarity‐based recognition [Eldridge et al., 2000; Fortin et al., 2004]. Strikingly, two very rare cases of epileptic patients with AP [Suriadi et al., 2015; Zatorre, 1989] demonstrated that the musical abilities and AP recognition remained intact after temporal lobectomy. In one case [Zatorre, 1989], the patient underwent an anterior temporal lobectomy in the left hemisphere that removed the amygdala and a sizable portion of hippocampal structures. In another case [Suriadi et al., 2015], the patient underwent a selective amygdalohippocampectomy in the right hemisphere to minimize damages in other temporal structures. The unimpaired AP listening after the operation implies that the hippocampus might not be necessary in retaining AP.

We suggest that the right anterior STP plays a key role in the pitch chroma extraction process, which corresponds to the early perceptual component of AP and should be a unique process only in the AP musicians but not in the non‐AP musicians. For further clarification, functional measurements (e.g., through fMRI) might be useful. If there exists a separate cortical module in the right PP that extracts pitch chroma, the region would be selectively sensitive to pitch chroma but not to pitch height. In other words, when an AP musician hears two tones an octave apart (e.g., C4 and C5), in the primary auditory cortex the activation peaks for the two tones should be distant along the tonotopic gradient [Moerel et al., 2012], whereas in the right PP the two peaks should be at the same position because of the identical pitch chromas of the two tones. Alternatively, instead of a geometrical (preferably geodesic) distance between brain sites, one could measure a high dimensional distance in a functional space as commonly utilized in multivariate pattern analysis methods such as a searchlight method [Haxby, 2012]. Under the same assumption, the functional distance amongst the local multivariate patterns in the right PP responding to the two tones with the same pitch chroma is expected to be closer than that in the primary auditory cortex. Such analyses will help to localize the AP template in the future.

### Intracortical Myelination and FDT

Further to some recent studies reporting in vivo measurements of myelin concentration and tonotopic organization in human auditory cortex [De Martino et al., 2014; Dick et al., 2012], we found a correlation between in vivo mapping of intracortical myelination in the right anterolateral HG extending to the lateral STG, which corresponds to Te3 in non‐primary auditory cortex [Morosan et al., 2001], and the frequency resolution in auditory perception of musicians for the first time. In the following paragraphs we briefly discuss how this finding relates to previous research.

The importance of the right lateral HG in pitch perception has been established in a number of functional and lesion studies in human and non‐human primate subjects [Bendor and Wang, 2005; Norman‐Haignere et al., 2013; Patterson et al., 2002; Penagos et al., 2004]. In marmoset monkeys, pitch‐selective neurons were found near the anterolateral border of the primary auditory cortex [Bendor and Wang, 2005]. In humans, pitch‐sensitive regions were also localized in the anterolateral part of the primary auditory cortex [Norman‐Haignere et al., 2013; Patterson et al., 2002]. Another human fMRI study, in which pitch salience was manipulated while controlling for temporal regularity of auditory stimuli, found a correlate of pitch salience at the anterolateral end of HG [Penagos et al., 2004]. Moreover, microelectrode recordings from bilateral HG in epileptic patients showed that responses of single neurons are selectively tuned with a very fine frequency resolution (frequency ratio of about 3%), which corresponds to the just‐noticeable difference in the untrained normal population [Bitterman et al., 2008].

In addition, greater involvement of the right hemisphere, compared to the left hemisphere, in pitch perception [Zatorre, 1998] has been further demonstrated in human studies using magnetoencephalography (MEG) [Patel and Balaban, 2001; Schneider et al., 2002], positron emission tomography (PET) [Zatorre, 1998], and fMRI [Zatorre and Belin, 2001]. Even more strikingly, human lesion studies showed that resection of the right HG greatly worsened the pitch discrimination ability, whereas it remained intact when only the left HG was removed [Johnsrude et al., 2000; Russell and Golfinos, 2003]. More recently, Coffey et al. demonstrated that the frequency‐locked auditory response (estimated from MEG source reconstruction) in the right auditory cortex was inversely correlated with a frequency discrimination threshold across individuals whereas such a relationship was not found in the left auditory cortex [Coffey et al., 2016]. There are also some morphological findings supporting the role of the right HG in pitch discrimination. Schneider et al. reported increased grey matter volume related to pitch discrimination performance [Schneider et al., 2002], albeit at a much coarser frequency resolution (>1 semitone) compared to the present study. In this context, our finding of a correlation between the cortical myelination in the right lateral HG and FDT supports the crucial role of the right lateral HG in pitch extraction and may provide an insight into the neural implementation of this process.

The separate locations of pitch chroma identification and fine pitch frequency discrimination in the right auditory cortex, which is specialized to spectral as opposed to temporal processing according to some theories [Poeppel, 2003], in terms of local cortical myelination (which might reflect local processing capabilities) highlights the distinctness of the underlying mechanisms of both faculties.

### Limitation

In the current dataset, there was a mismatch between the AP and non‐AP groups in terms of ethnicity (more Asian musicians than European musicians in the AP group), which was rooted in recruiting difficulties. However, we did demonstrate that the current findings with respect to AP could not be due to confounding influence of ethnicity.

The question remains as to whether there are some specific differences in cortical myelination that could be attributed to the ethnic background of the subjects. Although up to now no study has reported ethnic differences in cortical myelination, differences in cortical thickness and GM local volume were found in various areas of the cortex; (frontal, parietal, median temporal, and polymodal association cortices [Chee et al., 2011]. However, as discussed earlier, cortical thickness and myelination within the cortex may not be necessarily related. Although we did find effect of ethnicity in our current data (see Supporting Information for the effects of demographic variables), a large‐scale study is needed to elucidate the effects of ethnicity and any possible interaction with AP.

## CONCLUSION

We found greater intracortical myelination in an area within the right PP in AP musicians compared to non‐AP musicians, which was spatially distinct from another area in the anterolateral HG that correlated with FDT. We argue that heavier myelination of local wiring may be beneficial to the preservation of precise representations of pitch chroma after the acquisition of AP. Because it is known that the identity of an auditory object is processed along the ventral auditory pathway including the anterior STP, we suggest that the pitch chroma may be processed as an identifiable object property in AP musicians. Our findings indicate that the extraction of pitch chroma might occur in the anterior STP; however, further studies based on structural and functional brain imaging are needed to clarify whether and to what extent that region is merely a part of a wider network. Moreover, the localization of distinct cortical myelination related to pitch chroma identification and frequency discrimination in the right hemisphere is in favor of theories that postulate specialization of the left and right hemispheres in temporal and spectral processing, respectively.

## Supporting information

Supporting Information

## References

[hbm23254-bib-0001] Akiva‐Kabiri L , Henik A (2012): A unique asymmetrical stroop effect in absolute pitch possessors. Exp Psychol 59:272–278. 22617316 10.1027/1618-3169/a000153

[hbm23254-bib-0002] Altmann CF , Bledowski C , Wibral M , Kaiser J (2007): Processing of location and pattern changes of natural sounds in the human auditory cortex. Neuroimage 35:1192–1200. 17320413 10.1016/j.neuroimage.2007.01.007

[hbm23254-bib-0003] Arnott SR , Binns MA , Grady CL , Alain C (2004): Assessing the auditory dual‐pathway model in humans. Neuroimage 22:401–408. 15110033 10.1016/j.neuroimage.2004.01.014

[hbm23254-bib-0004] Ashburner J , Friston KJ (2000): Voxel‐based morphometry ‐ The methods. Neuroimage 11:805–821. 10860804 10.1006/nimg.2000.0582

[hbm23254-bib-0005] Baharloo S , Johnston PA , Service SK , Gitschier J , Freimer NB (1998): Absolute pitch: An approach for identification of genetic and nongenetic components. Am J Hum Genet 62:224–231. 9463312 10.1086/301704 PMC1376881

[hbm23254-bib-0006] Baharloo S , Service SK , Risch N , Gitschier J , Freimer NB (2000): Familial aggregation of absolute pitch. Am J Hum Genet 67:755–758. 10924408 10.1086/303057 PMC1287535

[hbm23254-bib-0007] Barrett DJ , Hall DA (2006): Response preferences for “what” and “where” in human non‐primary auditory cortex. Neuroimage 32:968–977. 16733092 10.1016/j.neuroimage.2006.03.050

[hbm23254-bib-0008] Bavelier D , Levi DM , Li RW , Dan Y , Hensch TK (2010): Removing brakes on adult brain plasticity: From molecular to behavioral interventions. J Neurosci 30:14964–14971. 21068299 10.1523/JNEUROSCI.4812-10.2010 PMC2992973

[hbm23254-bib-0009] Belin P , Zatorre RJ , Lafaille P , Ahad P , Pike B (2000): Voice‐selective areas in human auditory cortex. Nature 403:309–312. 10659849 10.1038/35002078

[hbm23254-bib-0010] Bendor D , Wang X (2005): The neuronal representation of pitch in primate auditory cortex. Nature 436:1161–1165. 16121182 10.1038/nature03867 PMC1780171

[hbm23254-bib-0011] Bermudez P , Lerch JP , Evans AC , Zatorre RJ (2009): Neuroanatomical correlates of musicianship as revealed by cortical thickness and voxel‐based morphometry. Cereb Cortex 19:1583–1596. 19073623 10.1093/cercor/bhn196

[hbm23254-bib-0012] Bermudez P , Zatorre RJ (2009a): The absolute pitch mind continues to reveal itself. J Biol 8:75. 19725935 10.1186/jbiol171 PMC2776913

[hbm23254-bib-0013] Bermudez P , Zatorre RJ (2009b): A distribution of absolute pitch ability as revealed by computerized testing. Music Percept 27:89–101.

[hbm23254-bib-0014] Binczak S , Eilbeck JC , Scott AC (2001): Ephaptic coupling of myelinated nerve fibers. Phys D 148:159–174.

[hbm23254-bib-0015] Bitterman Y , Mukamel R , Malach R , Fried I , Nelken I (2008): Ultra‐fine frequency tuning revealed in single neurons of human auditory cortex. Nature 451:197–201. 18185589 10.1038/nature06476 PMC2676858

[hbm23254-bib-0016] Blackmon K , Halgren E , Barr WB , Carlson C , Devinsky O , DuBois J , Quinn BT , French J , Kuzniecky R , Thesen T (2011): Individual differences in verbal abilities associated with regional blurring of the left gray and white matter boundary. J Neurosci 31:15257–15263. 22031871 10.1523/JNEUROSCI.3039-11.2011 PMC3865435

[hbm23254-bib-0017] Chee MWL , Zheng H , Goh JOS , Park D , Sutton BP (2011): Brain Structure in young and old East Asians and Westerners: Comparisons of structural volume and cortical thickness. J Cogn Neurosci 23:1065–1079. 20433238 10.1162/jocn.2010.21513 PMC3361742

[hbm23254-bib-0018] Chung MK , Robbins S , Evans AC (2005): Unified statistical approach to cortical thickness analysis. Inf Process Med Imaging 19:627–638. 17354731 10.1007/11505730_52

[hbm23254-bib-0019] Clarke S , Morosan P. 2012 Architecture, connectivity, and transmitter receptors of human auditory cortex In: Poeppel D, Overath T, Popper AN, Fay RR, editors. The Human Auditory Cortex. Springer pp 11–38.

[hbm23254-bib-0020] Coffey EB , Herholz SC , Chepesiuk AM , Baillet S , Zatorre RJ (2016): Cortical contributions to the auditory frequency‐following response revealed by MEG. Nat Commun 7. 10.1038/ncomms11070 PMC4820836 27009409

[hbm23254-bib-0021] Dale AM , Fischl B , Sereno MI (1999): Cortical surface‐based analysis ‐ I. Segmentation and surface reconstruction. Neuroimage 9:179–194. 9931268 10.1006/nimg.1998.0395

[hbm23254-bib-0022] De Martino F , Moerel M , Xu J , van de Moortele PF , Ugurbil K , Goebel R , Yacoub E , Formisano E. (2014): High‐resolution mapping of myeloarchitecture In vivo: localization of auditory areas in the human brain. Cereb Cortex. 10.1093/cercor/bhu150 PMC4585494 24994817

[hbm23254-bib-0023] Deutsch D. 2013 The Psychology of Music. Amsterdam: Academic Press.

[hbm23254-bib-0024] Deutsch D , Henthorn T (2004): Absolute pitch, speech, and tone language: Some experiments and a proposed framework. Music Percept 21:339–356.

[hbm23254-bib-0025] Dick F , Tierney AT , Lutti A , Josephs O , Sereno MI , Weiskopf N (2012): In vivo functional and myeloarchitectonic mapping of human primary auditory areas. J Neurosci 32:16095–16105. 23152594 10.1523/JNEUROSCI.1712-12.2012 PMC3531973

[hbm23254-bib-0026] Dohn A , Garza‐Villarreal EA , Chakravarty MM , Hansen M , Lerch JP , Vuust P (2015): Gray‐ and white‐matter anatomy of absolute pitch possessors. Cereb Cortex 25:1379–1388. 24304583 10.1093/cercor/bht334

[hbm23254-bib-0027] Eldridge LL , Knowlton BJ , Furmanski CS , Bookheimer SY , Engel SA (2000): Remembering episodes: a selective role for the hippocampus during retrieval. Nat Neurosci 3:1149–1152. 11036273 10.1038/80671

[hbm23254-bib-0028] Fischl B (2012): FreeSurfer. Neuroimage 62:774–781. 22248573 10.1016/j.neuroimage.2012.01.021 PMC3685476

[hbm23254-bib-0029] Fortin NJ , Wright SP , Eichenbaum H (2004): Recollection‐like memory retrieval in rats is dependent on the hippocampus. Nature 431:188–191. 15356631 10.1038/nature02853 PMC4053162

[hbm23254-bib-0030] Gervain J , Vines BW , Chen LM , Seo RJ , Hensch TK , Werker JF , Young AH (2013): Valproate reopens critical‐period learning of absolute pitch. Front Syst Neurosci 7:102. 24348349 10.3389/fnsys.2013.00102 PMC3848041

[hbm23254-bib-0031] Glasser MF , Van Essen DC (2011): Mapping Human Cortical Areas In Vivo Based on Myelin Content as Revealed by T1‐ and T2‐Weighted MRI. J Neurosci 31:11597–11616. 21832190 10.1523/JNEUROSCI.2180-11.2011 PMC3167149

[hbm23254-bib-0032] Good CD , Johnsrude I , Ashburner J , Henson RNA , Friston KJ , Frackowiak RSJ (2001a): Cerebral asymmetry and the effects of sex and handedness on brain structure: A voxel‐based morphometric analysis of 465 normal adult human brains. Neuroimage 14:685–700. 11506541 10.1006/nimg.2001.0857

[hbm23254-bib-0033] Good CD , Johnsrude IS , Ashburner J , Henson RNA , Friston KJ , Frackowiak RSJ (2001b): A voxel‐based morphometric study of ageing in 465 normal adult human brains. Neuroimage 14:21–36. 11525331 10.1006/nimg.2001.0786

[hbm23254-bib-0034] Griffiths TD , Hall DA (2012): Mapping pitch representation in neural ensembles with fMRI. J Neurosci 32:13343–13347. 23015424 10.1523/JNEUROSCI.3813-12.2012 PMC6621372

[hbm23254-bib-0035] Griffiths TD , Warren JD (2004): What is an auditory object? Nat Rev Neurosci 5:887–892. 15496866 10.1038/nrn1538

[hbm23254-bib-0036] Grydeland H , Walhovd KB , Tamnes CK , Westlye LT , Fjell AM (2013): Intracortical myelin links with performance variability across the human lifespan: Results from T1‐and T2‐weighted MRI myelin mapping and diffusion tensor imaging. J Neurosci 33:18618–18630. 24259583 10.1523/JNEUROSCI.2811-13.2013 PMC6618798

[hbm23254-bib-0037] Grydeland H , Westlye LT , Walhovd KB , Fjell AM. (2015): Intracortical posterior cingulate myelin content relates to error processing: Results from T1‐ and T2‐weighted MRI myelin mapping and electrophysiology in healthy adults. Cereb Cortex. 10.1093/cercor/bhv065 25840423

[hbm23254-bib-0038] Gur RC , Turetsky BI , Matsui M , Yan M , Bilker W , Hughett P , Gur RE (1999): Sex differences in brain gray and white matter in healthy young adults: Correlations with cognitive performance. J Neurosci 19:4065–4072. 10234034 10.1523/JNEUROSCI.19-10-04065.1999 PMC6782697

[hbm23254-bib-0039] Hart HC , Palmer AR , Hall DA (2004): Different areas of human non‐primary auditory cortex are activated by sounds with spatial and nonspatial properties. Hum Brain Mapp 21:178–190. 14755837 10.1002/hbm.10156 PMC6872110

[hbm23254-bib-0040] Hashim E , Rowley CD , Grad S , Bock NA (2015): Patterns of myeloarchitecture in lower limb amputees: an MRI study. Front Neurosci 9:15. 25698916 10.3389/fnins.2015.00015 PMC4318335

[hbm23254-bib-0041] Haxby JV (2012): Multivariate pattern analysis of fMRI: The early beginnings. Neuroimage 62:852–855. 22425670 10.1016/j.neuroimage.2012.03.016 PMC3389290

[hbm23254-bib-0042] Itoh K , Suwazono S , Arao H , Miyazaki K , Nakada T (2005): Electrophysiological correlates of absolute pitch and relative pitch. Cereb Cortex 15:760–769. 15371294 10.1093/cercor/bhh177

[hbm23254-bib-0043] Johnsrude IS , Penhune VB , Zatorre RJ (2000): Functional specificity in the right human auditory cortex for perceiving pitch direction. Brain 123:155–163. 10611129 10.1093/brain/123.1.155

[hbm23254-bib-0044] Keenan JP , Thangaraj V , Halpern AR , Schlaug G (2001): Absolute pitch and planum temporale. Neuroimage 14:1402–1408. 11707095 10.1006/nimg.2001.0925

[hbm23254-bib-0045] Kusmierek P , Rauschecker JP (2009): Functional specialization of medial auditory belt cortex in the alert rhesus monkey. J Neurophysiol 102:1606–1622. 19571201 10.1152/jn.00167.2009 PMC2746772

[hbm23254-bib-0046] Levitin DJ , Rogers SE (2005): Absolute pitch: perception, coding, and controversies. Trends Cogn Sci 9:26–33. 15639438 10.1016/j.tics.2004.11.007

[hbm23254-bib-0047] Luders E , Gaser C , Jancke L , Schlaug G (2004): A voxel‐based approach to gray matter asymmetries. Neuroimage 22:656–664. 15193594 10.1016/j.neuroimage.2004.01.032

[hbm23254-bib-0048] Lutti A , Dick F , Sereno MI , Weiskopf N (2014): Using high‐resolution quantitative mapping of R1 as an index of cortical myelination. Neuroimage 93:176–188. 23756203 10.1016/j.neuroimage.2013.06.005

[hbm23254-bib-0049] Marques JP , Gruetter R (2013): New developments and applications of the MP2RAGE sequence–focusing the contrast and high spatial resolution R1 mapping. PLoS One 8:e69294. 23874936 10.1371/journal.pone.0069294 PMC3712929

[hbm23254-bib-0050] Marques JP , Kober T , Krueger G , van der Zwaag W , Van de Moortele PF , Gruetter R (2010): MP2RAGE, a self bias‐field corrected sequence for improved segmentation and T1‐mapping at high field. Neuroimage 49:1271–1281. 19819338 10.1016/j.neuroimage.2009.10.002

[hbm23254-bib-0051] McGee AW , Yang Y , Fischer QS , Daw NW , Strittmatter SM (2005): Experience‐driven plasticity of visual cortex limited by myelin and nogo receptor. Science 309:2222–2226. 16195464 10.1126/science.1114362 PMC2856689

[hbm23254-bib-0052] Micheyl C , Delhommeau K , Perrot X , Oxenham AJ (2006): Influence of musical and psychoacoustical training on pitch discrimination. Hear Res 219:36–47. 16839723 10.1016/j.heares.2006.05.004

[hbm23254-bib-0053] Miyazaki K (1988): Musical pitch identification by absolute pitch possessors. Percept Psychophys 44:501–512. 3200669 10.3758/bf03207484

[hbm23254-bib-0054] Miyazaki K (1990): The speed of musical pitch identification by absolute‐pitch possessors. Music Percept 8:177–188. 10.3758/bf03207484 3200669

[hbm23254-bib-0055] Miyazaki K (2004a): How well do we understand absolute pitch? Acoust Sci Technol 25:426–432.

[hbm23254-bib-0056] Miyazaki K (2004b): Recognition of transposed melodies by absolute‐pitch possessors. Jpn Psychol Res 46:270–282.

[hbm23254-bib-0057] Miyazaki K , Makomaska S , Rakowski A (2012): Prevalence of absolute pitch: A comparison between Japanese and Polish music students. J Acoust Soc Am 132:3484–3493. 23145628 10.1121/1.4756956

[hbm23254-bib-0058] Miyazaki K , Ogawa Y (2006): Learning absolute pitch by children: A cross‐sectional study. Music Percept 24:63–78.

[hbm23254-bib-0059] Miyazaki K (1989): Absolute pitch identification: Effects of timbre and pitch region. Music Percept 7:1–14.

[hbm23254-bib-0060] Moerel M , De Martino F , Formisano E (2012): Processing of natural sounds in human auditory cortex: Tonotopy, spectral tuning, and relation to voice sensitivity. J Neurosci 32:14205–14216. 23055490 10.1523/JNEUROSCI.1388-12.2012 PMC6622378

[hbm23254-bib-0061] Morosan P , Rademacher J , Schleicher A , Amunts K , Schormann T , Zilles K (2001): Human primary auditory cortex: Cytoarchitectonic subdivisions and mapping into a spatial reference system. Neuroimage 13:684–701. 11305897 10.1006/nimg.2000.0715

[hbm23254-bib-0062] Nieuwenhuys R (2013): The myeloarchitectonic studies on the human cerebral cortex of the Vogt‐Vogt school, and their significance for the interpretation of functional neuroimaging data. Brain Struct Funct 218:303–352. 23076375 10.1007/s00429-012-0460-z

[hbm23254-bib-0063] Norman‐Haignere S , Kanwisher N , McDermott JH (2013): Cortical pitch regions in humans respond primarily to resolved harmonics and are located in specific tonotopic regions of anterior auditory cortex. J Neurosci 33:19451–19469. 24336712 10.1523/JNEUROSCI.2880-13.2013 PMC3916670

[hbm23254-bib-0064] Oechslin MS , Imfeld A , Loenneker T , Meyer M , Jancke L (2009): The plasticity of the superior longitudinal fasciculus as a function of musical expertise: A diffusion tensor imaging study. Front Hum Neurosci 3:76. 20161812 10.3389/neuro.09.076.2009 PMC2821183

[hbm23254-bib-0065] Ohnishi T , Matsuda H , Asada T , Aruga M , Hirakata M , Nishikawa M , Katoh A , Imabayashi E (2001): Functional anatomy of musical perception in musicians. Cereb Cortex 11:754–760. 11459765 10.1093/cercor/11.8.754

[hbm23254-bib-0066] Oldfield RC (1971): The assessment and analysis of handedness: The Edinburgh inventory. Neuropsychologia 9:97–113. 5146491 10.1016/0028-3932(71)90067-4

[hbm23254-bib-0067] Patel AD , Balaban E (2001): Human pitch perception is reflected in the timing of stimulus‐related cortical activity. Nat Neurosci 4:839–844. 11477431 10.1038/90557

[hbm23254-bib-0068] Patterson RD , Uppenkamp S , Johnsrude IS , Griffiths TD (2002): The processing of temporal pitch and melody information in auditory cortex. Neuron 36:767–776. 12441063 10.1016/s0896-6273(02)01060-7

[hbm23254-bib-0069] Penagos H , Melcher JR , Oxenham AJ (2004): A neural representation of pitch salience in nonprimary human auditory cortex revealed with functional magnetic resonance imaging. J Neurosci 24:6810–6815. 15282286 10.1523/JNEUROSCI.0383-04.2004 PMC1794212

[hbm23254-bib-0070] Poeppel D (2003): The analysis of speech in different temporal integration windows: Cerebral lateralization as 'asymmetric sampling in Time'. Speech Commun 41:245–255.

[hbm23254-bib-0071] Rauschecker JP (2015): Auditory and visual cortex of primates: A comparison of two sensory systems. Eur J Neurosci 41:579–585. 25728177 10.1111/ejn.12844 PMC4347938

[hbm23254-bib-0072] Rivier F , Clarke S (1997): Cytochrome oxidase, acetylcholinesterase, and NADPH‐diaphorase staining in human supratemporal and insular cortex: Evidence for multiple auditory areas. Neuroimage 6:288–304. 9417972 10.1006/nimg.1997.0304

[hbm23254-bib-0073] Russell SM , Golfinos JG (2003): Amusia following resection of a Heschl gyrus glioma ‐ Case report. J Neurosurg 98:1109–1112. 12744373 10.3171/jns.2003.98.5.1109

[hbm23254-bib-0074] Schlaug G , Jancke L , Huang YX , Steinmetz H (1995): In‐vivo evidence of structural brain asymmetry in musicians. Science 267:699–701. 7839149 10.1126/science.7839149

[hbm23254-bib-0075] Schneider P , Scherg M , Dosch HG , Specht HJ , Gutschalk A , Rupp A (2002): Morphology of Heschl's gyrus reflects enhanced activation in the auditory cortex of musicians. Nat Neurosci 5:688–694. 12068300 10.1038/nn871

[hbm23254-bib-0076] Schulze K , Mueller K , Koelsch S (2013): Auditory stroop and absolute pitch: An fMRI study. Hum Brain Mapp 34:1579–1590. 22359341 10.1002/hbm.22010 PMC6870281

[hbm23254-bib-0077] Shafee R , Buckner RL , Fischl B (2015): Gray matter myelination of 1555 human brains using partial volume corrected MRI images. Neuroimage 105:473–485. 25449739 10.1016/j.neuroimage.2014.10.054 PMC4262571

[hbm23254-bib-0078] Shen S , Li J , Casaccia‐Bonnefil P (2005): Histone modifications affect timing of oligodendrocyte progenitor differentiation in the developing rat brain. J Cell Biol 169:577–589. 15897262 10.1083/jcb.200412101 PMC2171688

[hbm23254-bib-0079] Siegel JA (1974): Sensory and verbal coding strategies in subjects with absolute pitch. J Exp Psychol 103:37–44. 4423196 10.1037/h0036844

[hbm23254-bib-0080] Sigalovsky IS , Fischl B , Melcher JR (2006): Mapping an intrinsic MR property of gray matter in auditory cortex of living humans: a possible marker for primary cortex and hemispheric differences. Neuroimage 32:1524–1537. 16806989 10.1016/j.neuroimage.2006.05.023 PMC1839042

[hbm23254-bib-0081] Suriadi MM , Usui K , Tottori T , Terada K , Fujitani S , Umeoka S , Usui N , Baba K , Matsuda K , Inoue Y (2015): Preservation of absolute pitch after right amygdalohippocampectomy for a pianist with TLE. Epilepsy Behav 42:14–17. 25499156 10.1016/j.yebeh.2014.10.025

[hbm23254-bib-0082] Takeuchi AH , Hulse SH (1993): Absolute pitch. Psychol Bull 113:345–361. 8451339 10.1037/0033-2909.113.2.345

[hbm23254-bib-0083] Tardif E , Clarke S (2001): Intrinsic connectivity of human auditory areas: A tracing study with DiI. Eur J Neurosci 13:1045–1050. 11264678 10.1046/j.0953-816x.2001.01456.x

[hbm23254-bib-0084] Thambisetty M , Wan J , Carass A , An Y , Prince JL , Resnick SM (2010): Longitudinal changes in cortical thickness associated with normal aging. Neuroimage 52:1215–1223. 20441796 10.1016/j.neuroimage.2010.04.258 PMC2910226

[hbm23254-bib-0085] Tian B , Reser D , Durham A , Kustov A , Rauschecker JP (2001): Functional specialization in rhesus monkey auditory cortex. Science 292:290–293. 11303104 10.1126/science.1058911

[hbm23254-bib-0086] von Kriegstein K , Warren JD , Ives DT , Patterson RD , Griffiths TD (2006): Processing the acoustic effect of size in speech sounds. Neuroimage 32:368–375. 16644240 10.1016/j.neuroimage.2006.02.045

[hbm23254-bib-0087] Wallace MN , Johnston PW , Palmer AR (2002): Histochemical identification of cortical areas in the auditory region of the human brain. Exp Brain Res 143:499–508. 11914796 10.1007/s00221-002-1014-z

[hbm23254-bib-0088] Warren JD , Griffiths TD (2003): Distinct mechanisms for processing spatial sequences and pitch sequences in the human auditory brain. J Neurosci 23:5799–5804. 12843284 10.1523/JNEUROSCI.23-13-05799.2003 PMC6741275

[hbm23254-bib-0089] Wengenroth M , Blatow M , Heinecke A , Reinhardt J , Stippich C , Hofmann E , Schneider P (2014): Increased volume and function of right auditory cortex as a marker for absolute pitch. Cereb Cortex 24:1127–1137. 23302811 10.1093/cercor/bhs391

[hbm23254-bib-0090] Wilson SJ , Lusher D , Wan CY , Dudgeon P , Reutens DC (2009): The neurocognitive components of pitch processing: Insights from absolute pitch. Cereb Cortex 19:724–732. 18663250 10.1093/cercor/bhn121 PMC2638817

[hbm23254-bib-0091] Worsley KJ , Evans AC , Marrett S , Neelin P (1992): A three‐dimensional statistical analysis for CBF activation studies in human brain. J Cereb Blood Flow Metab 12:900–918. 1400644 10.1038/jcbfm.1992.127

[hbm23254-bib-0092] Worsley KJ , Taylor JE , Carbonell F , Chung MK , Duerden E , Bernhardt B , Lyttelton O , Boucher M , Evans AC (2009): SurfStat: A Matlab toolbox for the statistical analysis of univariate and multivariate surface and volumetric data using linear mixed effects models and random field theory. Neuroimage 47:S102.

[hbm23254-bib-0093] Zatorre RJ (1989): Intact absolute pitch ability after left temporal lobectomy. Cortex 25:567–580. 2612176 10.1016/s0010-9452(89)80018-8

[hbm23254-bib-0094] Zatorre RJ (1998): Functional specialization of human auditory cortex for musical processing. Brain 121:1817–1818. 9798739 10.1093/brain/121.10.1817

[hbm23254-bib-0095] Zatorre RJ , Belin P (2001): Spectral and temporal processing in human auditory cortex. Cereb Cortex 11:946–953. 11549617 10.1093/cercor/11.10.946

[hbm23254-bib-0096] Zatorre RJ , Perry DW , Beckett CA , Westbury CF , Evans AC (1998): Functional anatomy of musical processing in listeners with absolute pitch and relative pitch. Proc Natl Acad Sci USA 95:3172–3177. 9501235 10.1073/pnas.95.6.3172 PMC19714

[hbm23254-bib-0097] Zatorre RJ , Zarate JM. (2012): Cortical processing of music. 43:261–294.

