# Supplementary material for: Intracortical myelination in musicians with absolute pitch: Quantitative morphometry using 7‐T MRI
Source: Hum Brain Mapp. 2016 May 10;37(10):3486–501. doi: 10.1002/hbm.23254 (PMC5084814; doi:10.1002/hbm.23254)
Supplement: Supplementary file 1 — Supporting Information [file HBM-37-3486-s001.pdf]

Supporting information

Title: Intracortical myelination in musicians with absolute pitch: quantitative morphometry using 7-T MRI

Authors: Seung-Goo Kim, Thomas R. Knösche
Affiliation: Max-Planck Institute for Human Cognitive and Brain Sciences, Leipzig, Germany

Corresponding author: Seung-Goo Kim
Postal address: Stephanstraße 1A, 04103 Leipzig, Germany
Phone: +49 341 9940 2618
Facsimile: +49 341 9940 2624
Email: sol@snu.ac.kr

Detailed description of image processing

Skull-stripping of MP2RAGE image.

In ultra-high (≥ 7T) static magnetic fields, T1-weighted (T1w) imaging is highly affected by inhomogeneities in the transmit field. To minimize such biases, the T1w image in the MP2RAGE sequence is created by combining two inversion images (Marques et al., 2010). This results in uniform tissue contrast over the whole brain, but it also amplifies noise outside the head and in areas adjacent to the brain, which somewhat hampers the use of common image processing tools that are optimized for conventional T1w images at lower field strength (≤3T) MRIs. Thus, we performed brain extraction in two steps: (1) Brain Extraction Tool (BET) in FMRIB Software Library (FSL; http://fsl.fmrib.ox.ac.uk/fsl/fslwiki/) (Jenkinson et al., 2012) was applied to the second inversion image, which has a similar contrast to proton density images, with a liberal fractional intensity threshold of 0.1, in order to create a rough brain mask that discards background noise outside the head. However, the noise in the dura mater still remains after BET. (2) We fed the brain mask to the Voxel-Based Morphometry Toolbox 8 (VBM8; http://www.neuro.uni-jena.de/vbm/) on Statistic Parametric Mapping (SPM; http://www.fil.ion.ucl.ac.uk/spm/) (Ashburner, 2012) to obtain tissue segmentations of gray matter, white matter, and cerebrospinal fluid. Finally, the skull-stripped T1w image was created by masking the unbiased T1w image with the union of the three segmentations from VBM8. Extracted brain images were visually inspected and manually corrected if necessary.

Cortical surface construction and qR1 resampling

Cortical surfaces were reconstructed using FreeSurfer (http://freesurfer.net/) (Fischl, 2012) from the skull-stripped T1w image that was down-sampled to 1-mm isotropic resolution. The average number of vertices was about 140,000 per hemisphere, and the average lengths of edges were 0.99 ± 0.41 mm ('pial' surface, interface between the gray matter and cerebrospinal fluid) and 0.88 ± 0.25 mm ('white' surface, interface between the gray and white matter). Three layers between the pial and white surfaces were modeled by displacing vertices of the white surface along the outward normal vectors by the 25, 50, and 75% of cortical thicknesses. Subsequently, the qT1 image at 0.7-mm isovoxel resolution was directly sampled at the vertices of these 3 individual cortical surfaces using nearest

neighbor interpolation. The individual qT1 maps on native surfaces were then registered to a template mesh contained in FreeSurfer (called 'fsaverage' in MNI305 space) using spherical mapping that aligns local curvature. Finally, qR1 (= 1/qT1) values were computed from the surface-mapped qT1 values and bounded within a range between 0.25 and 10 s^{-1}, which corresponds to the qT1 range between 100 and 4000 msec.

Correlation between local curvature and cortical myelin

Spurious correlations between cortical myelin density, cortical thickness, and local curvature were reported from histological samples (Annese et al., 2004) and a large-scale in-vivo MRI dataset (Shafee et al., 2015). Those relationships are known to be negative (greater myelination in a thinner or more convex region of the cortex) (Shafee et al., 2015). To construct a grid system free of such a bias, Waehnert et al. (2014) implemented a novel layering method that preserves local volume so that the modeled layers mimic the physical bending process of cortical sheets. Although such layer modeling is highly attractive and worked better in ex-vivo images at 0.14-mm isotropic resolution, as shown in Waehnert et al. (2014), the result of the isovolumetric model was very similar to that of other methods, such as an isopotential model using the Laplacian or an equidistant model, suggesting that the benefit of the novel layering method would be limited in our dataset at 0.7-mm isotropic resolution. Thus, we used an equidistant model (i.e., equal thickness of 'layers' at each point) by fixed proportion of cortical thickness.

In the present data, we indeed found a negative correlation of qR1 values with curvature and thickness as previously reported in the literature (Dick et al., 2012; Lutti et al., 2014; Sereno et al., 2013). However, the effect sizes were very small and negligible (correlation between qR1 and curvature was -0.0058 ± 0.0026; correlation between qR1 and thickness was -0.0757 ± 0.0083). The significance level was very high ($p < 10^{-16}$), but this was mainly due to the very large number of measures (more than 140,000 vertices for each hemisphere). This also explains why the 'de-curved/de-thickened' qR1 values were very similar to the demeaned qR1 values in those studies (Dick et al., 2012; Sereno et al., 2013). Consequently, we did not use the geometrical covariates in our statistical analysis.

Effects of demographic variables

To determine whether covarying demographic variables is necessary, we tested effects of age, sex, and ethnicity using a very simple model as:

$$qR_1 = \beta_0 + D\beta_1 + \varepsilon \quad \text{(Eq. S1)}$$

where D is either age, sex, or ethnicity. We found significant effects over extensive areas including right inferior frontal gyrus, right anterior cingulate gyrus, and right superior temporal gyrus (age effect; **Figure S1**, upper row), right superior temporal sulcus (sex effect; Figure S1, middle row), and left planum temporale and supra marginal gyrus (ethnicity effect; **Figure S1**, lower row). Given the significant effects of demographic variables, we incorporated them as covariate terms in order to control possible confounding.

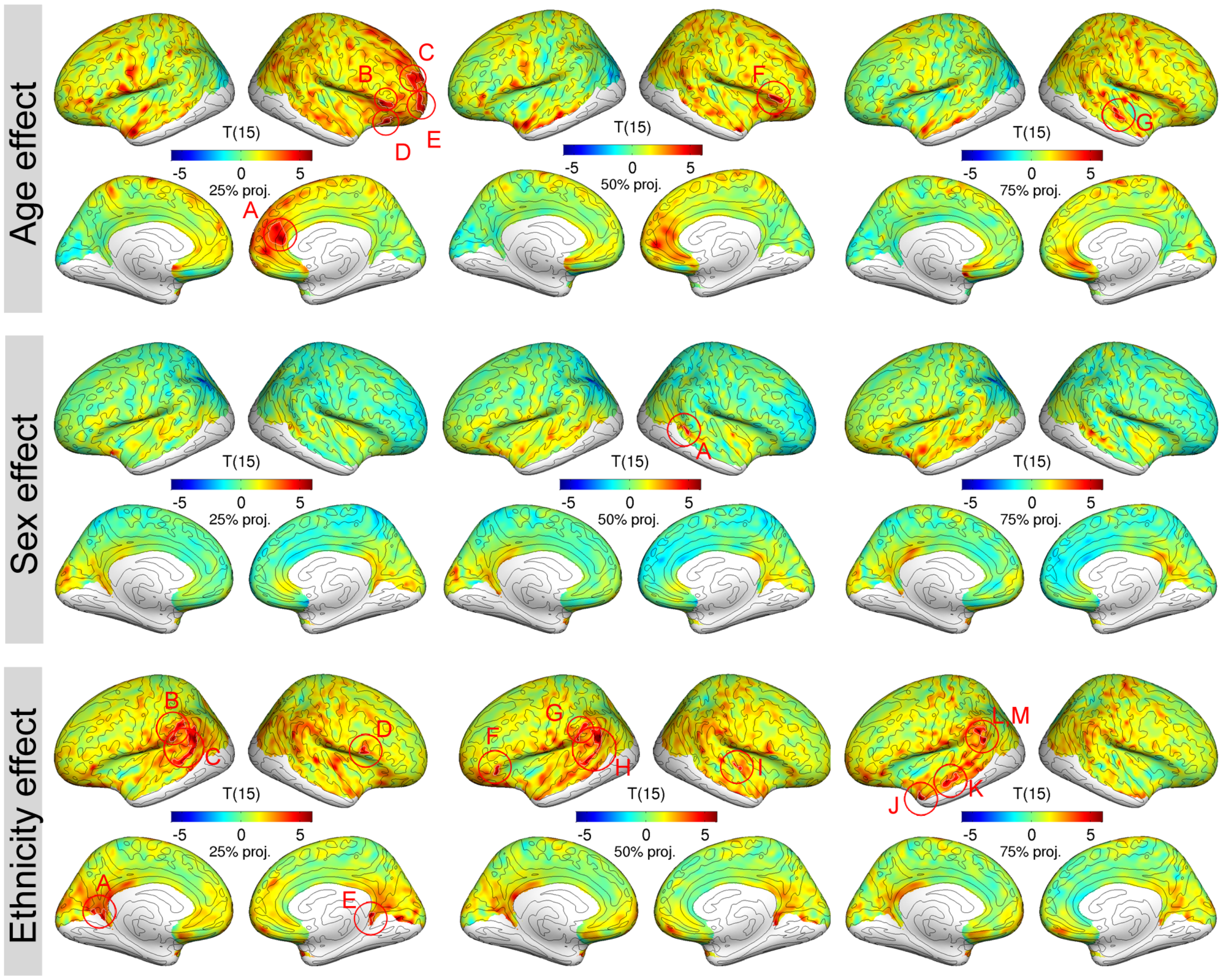

Figure S1. The effect of demographic variables (age, sex, and ethnicity) on quantitative longitudinal relaxation rate (qR1). T-statistic maps are given for each projection level. See **Table S-I** for details of significant clusters.

Table S-I. Significant clusters for the effect of demographic variables

	Side	Level*	Structure name	T (15)	p-value+	Area (mm²)	MNI-305 (x, y, z mm)		
Age effect									
A	Right	25%	Right anterior cingulate cortex	6.15	0.0037	308.8	1.6	42.6	9.2
B	Right	25%	Right triangular part of the interior frontal gyrus	5.18	0.0111	108.2	51.1	26.3	-0.5
C	Right	25%	Right middle frontal sulcus	4.99	0.0188	314.0	23.7	44.2	19.6
D	Right	25%	Right orbital gyri	6.24	0.0184	72.0	37.2	25.7	-22.4
E	Right	25%	Right fronto-marginal gyrus/sulcus	4.99	0.0055	241.9	24.1	50.7	2.0
F	Right	50%	Right orbital part of the interior frontal gyrus	5.93	0.0092	81.4	53.0	30.3	-2.6
G	Right	75%	Right superior temporal sulcus	6.26	0.0047	109.1	65.3	-11.1	-2.5
Sex effect									
A	Right	50%	Right superior temporal sulcus	4.71	0.0434	46.4	44.3	-38.8	-0.9

Ethnicity effect									
A	Left	25%	Left mid-post cingulate cortex	5.26	0.0064	123.4	-20.8	-53.2	-1.4
B	Left	25%	Left supramarginal gyrus	5.50	0.0069	185.7	-64.9	-34.1	36.2
C	Left	25%	Left planum temporale	6.06	0.0001	426.9	-64.6	-52.5	18.3
D	Right	25%	Right opercular part of the interior frontal gyrus	4.78	0.0356	85.8	55.1	14.2	13.9
E	Right	25%	Right parahippocampal gyrus	5.48	0.0088	86.6	6.1	-45.0	1.0
F	Left	50%	Left orbital part of the interior frontal gyrus	4.95	0.0249	46.6	-50.5	32.3	-11.3
G	Left	50%	Left supramarginal gyrus	4.94	0.0436	103.8	-64.9	-34.5	35.6
H	Left	50%	Left planum temporale	10.14	0.0001	308.4	-64.6	-52.5	18.3
I	Right	50%	Right planum polare	4.68	0.0460	57.1	46.5	-14.0	3.7
J	Left	75%	Left temporal pole	7.80	0.0375	68.2	-45.8	18.8	-30.5
K	Left	75%	Left superior temporal sulcus	5.29	0.0296	145.3	-53.9	-13.3	-16.7
L	Left	75%	Left planum temporale	6.46	0.0069	83.8	-60.5	-43.8	28.1
M	Left	75%	Left planum temporale	7.17	0.0072	48.1	-63.7	-53.8	15.5

* Projection level indicates its sampling point from gray matter and white matter interface along an outward normal vector by 25 % (deep), 50 % (middle), or 75 % (superficial) of cortical thickness. + P-values are corrected for multiple comparison correction at cluster level.

Controlling for the effect of ethnicity

Due to the imbalanced design of the current study in terms of ethnicity (i.e., 8 musicians with absolute pitch [AP] = 5 Asian musicians + 3 European musicians; 9 musicians without AP = 9 European musicians), one might wonder if it is possible to separate the effect of ethnicity from that of absolute pitch. To compare the bias in the current sample with that of an ideal scenario, a numerical simulation was carried out. As stated in the main text, the group effect was estimated using the model (Eq. S2):

$$qR_1 = \beta_0 + age\beta_1 + sex\beta_2 + ethnicity\beta_3 + AP\beta_4 + \varepsilon \quad \text{(Eq. S2)}$$

From the peak vertex at the middle depth (50% of cortical thickness) in the right planum polare, estimated coefficients were 0.4437 (interceptor), 0.0004 (age), 0.0040 (sex), -0.0093 (ethnicity), and 0.0169 (AP) s^{-1}. The standard deviation of the residuals was 0.0033 and the mean was 10^{-14} s^{-1}. The Kolmogorov-Smirnov test did not reject the null hypothesis that the distribution of residuals is normal (p = 0.845). Therefore, we used normal distribution to generate noise with the observed mean and standard deviation. Assuming the model (Eq.2) as well as effect size and noise level estimated from the current dataset as "ground truth", simulated datasets (k = 10,000) were generated for different design matrices varying the number of European AP musicians. Because the number of European AP musicians controls the separation between the ethnicity and AP variables, its change varies the collinearity between ethnicity and AP. The procedure of simulation is as follows:

1. Change the original design matrix X to make it more or less balanced by changing the ethnicity vector x'_e while leaving other vectors x_{-e} constant:

$$\mathbf{X} \leftarrow \left[\mathbf{x}_{-e} \quad \mathbf{x}'_{e}\right]$$

1.1. Generate data with assumed 'true' parameters of the effect size $\mathbf{b} = [\beta_0, \beta_1, \cdots, \beta_n]$, mean μ, and standard deviation σ of a normal distribution N:

$$y \leftarrow \mathbf{Xb} + N(\mu, \sigma^2)$$

1.2. Least-square-estimation (LSE) fitting where superscripted T means transposition and + means pseudoinverse:

$$\hat{\mathbf{b}} \leftarrow (\mathbf{X}^T\mathbf{X})^+ + \mathbf{X}^T y$$

1.3. Compute and store the relative error of estimation for the i-th regressor of interest:

$$e_{rel} \leftarrow (\hat{\beta}_i - \beta_i) / \beta_i$$

1.4. Repeat (1.1) to (1.4) for 10,000 times.

2. Repeat 1 for all possible design matrices.

Boxplots of relative errors of estimated effect sizes of AP over the correlation between ethnicity and AP are given in **Figure S2.** Because the generated noise was unbiased, the median of estimated $\hat{\beta}_i$ was very close to the true β_i in all cases. However, as the design becomes more imbalanced (i.e., greater correlation), the reliability of estimation decreases (i.e., more distant range between outliers and quantiles), and vice versa.

The result indicates that the reliability of estimating the effect size of AP under the current design (relative error= -0.09 ± 13.16 %; the blue squared boxplot in **Figure S2**) is still comparable to a perfectly balanced design (relative error= 0.05 ± 9.41 %, the leftmost boxplot in **Figure S2**) and much better than the worst case (relative error= -0.39 ± 21.39 %; the rightmost boxplot in **Figure S2**). Additionally, in terms of the minima and maxima of relative errors from 10,000 randomizations, the current design shows reasonable bounds ([-46.16, 49.06] %) with respect to the ideal one ([-38.01, 35.51] %) and certainly narrower than the worst one ([-79.38, 91.16] %).

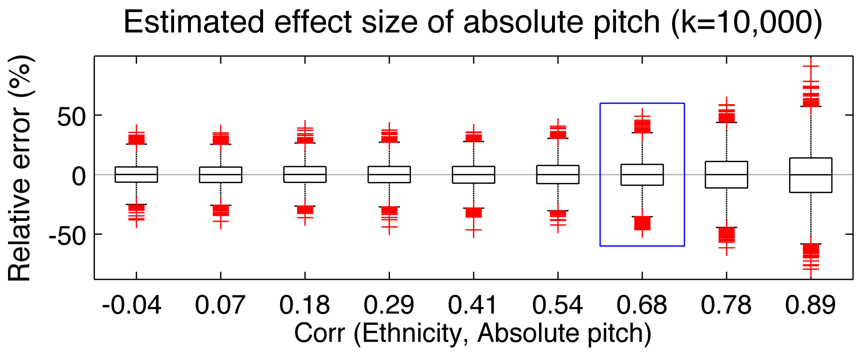

Figure S2. Relative errors in estimating effect size of absolute pitch over correlation between ethnicity and AP. The blue rectangle indicates the correlation between the two variables under the current design.

Effect of frequency discrimination threshold

We tested a GLM (Eq. 3 in the main text) as:

$$qR_1 = \beta_0 + age\beta_1 + sex\beta_2 + ethnicity\beta_3 + AP\beta_4 + (-\log FDT)\beta_5 + \varepsilon \quad \text{, (Eq. S3)}$$

where *FDT=F1/F0, F1* is the minimal frequency of a target that one can discriminate, and *F0* is the frequency of a reference. See Micheyl et al. (2006) for further details of the behavioral experiment. The results are given in **Figure S3** and **Table S-II.** The effect of frequency discrimination threshold (FDT) in the lateral region of the right Heschl's gyrus (HG) extending to the lateral superior temporal gyrus (STG) was found across all layers (**Figure S3**, A, B, C). Differences in other regions were found only in the superficial layer (75% of thickness from the white matter surface): i.e., in the opercular part of the right inferior frontal gyrus (**Figure S3**, E) and in the right lateral superior transverse gyrus of the parietal lobe (Figure S3, D).

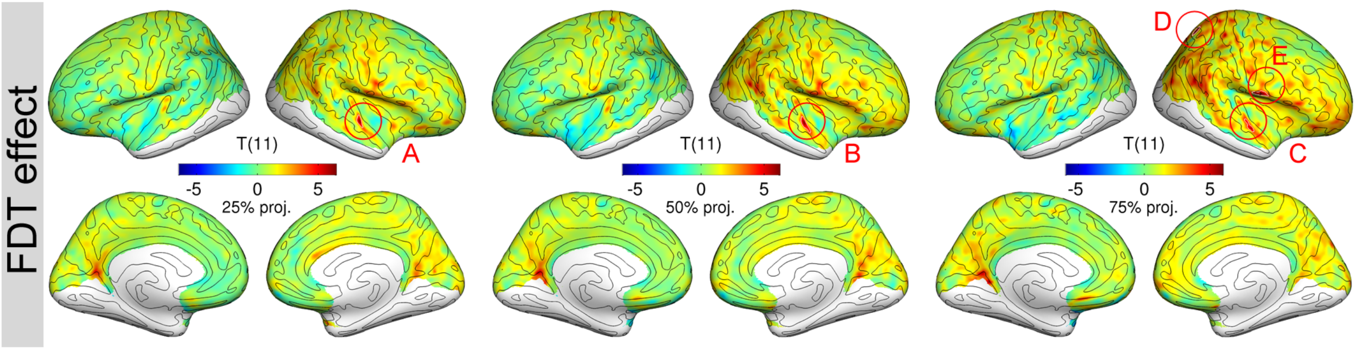

Figure S3. The effect of frequency discrimination threshold (FDT) on quantitative longitudinal relaxation rate (qR1). T-statistic maps are given for each projection level. See Table S-II for details of significant clusters.

Table S-II. Significant clusters for the effect of frequency discrimination threshold (FDT)

	Side	Level*	Structure name	T (11)	p-value+	Area (mm2)	MNI-305 (x, y, z mm)		
A	Right	25 %	Right transverse gyrus of Heschl	6.51	0.0331	46.8	59.9	-2.6	-0.8
B	Right	50 %	Right transverse gyrus of Heschl	6.26	0.0208	61.8	60.0	-1.4	-1.7
C	Right	75 %	Right lateral superior transverse gyrus	5.48	0.0240	37.3	60.1	2.6	-7.9
D	Right	75 %	Right intra-/trans-parietal sulci	6.01	0.0480	37.6	20.6	-59.1	51.2
E	Right	75 %	Right opercular part of the inferior frontal gyrus	5.78	0.0088	70.7	54.5	-5.5	6.4

* Projection level indicates its sampling point from gray matter and white matter interface along an outward normal vector by 25 % (deep), 50 % (middle), or 75 % (superficial) of cortical thickness. + P-values are corrected for multiple comparison correction at cluster level.

Subcortical white matter myelination

The main findings of altered cortical myelination in the anterior part of the right supratemporal plane (STP) that related to absolute pitch (AP) performance could either be due to long-range connections from/to the anterior STP or due to intracortical connections within that region. If increased myelination was also found in the white matter underneath the anterior STP, the additional cortical myelin in the AP musicians could be interpreted as part of a long-range connection. However, qR1 mapping is not an optimal choice to investigate white matter myelination compared to magnetization transfer imaging or diffusion-weighted imaging. Here, we only explored such a possibility from qR1 data, but it should be further examined using white matter myelin imaging in a future study.

We sampled qR1 values from subcortical white matter at 25, 50, and 75 % of cortical thickness along the inward normal vectors pointing to the white matter. All sampling points were visually inspected (see **Figure S4** for an example) and computationally confirmed that coordinates of all sampling points are confined in the white matter mask in 3-D volume.

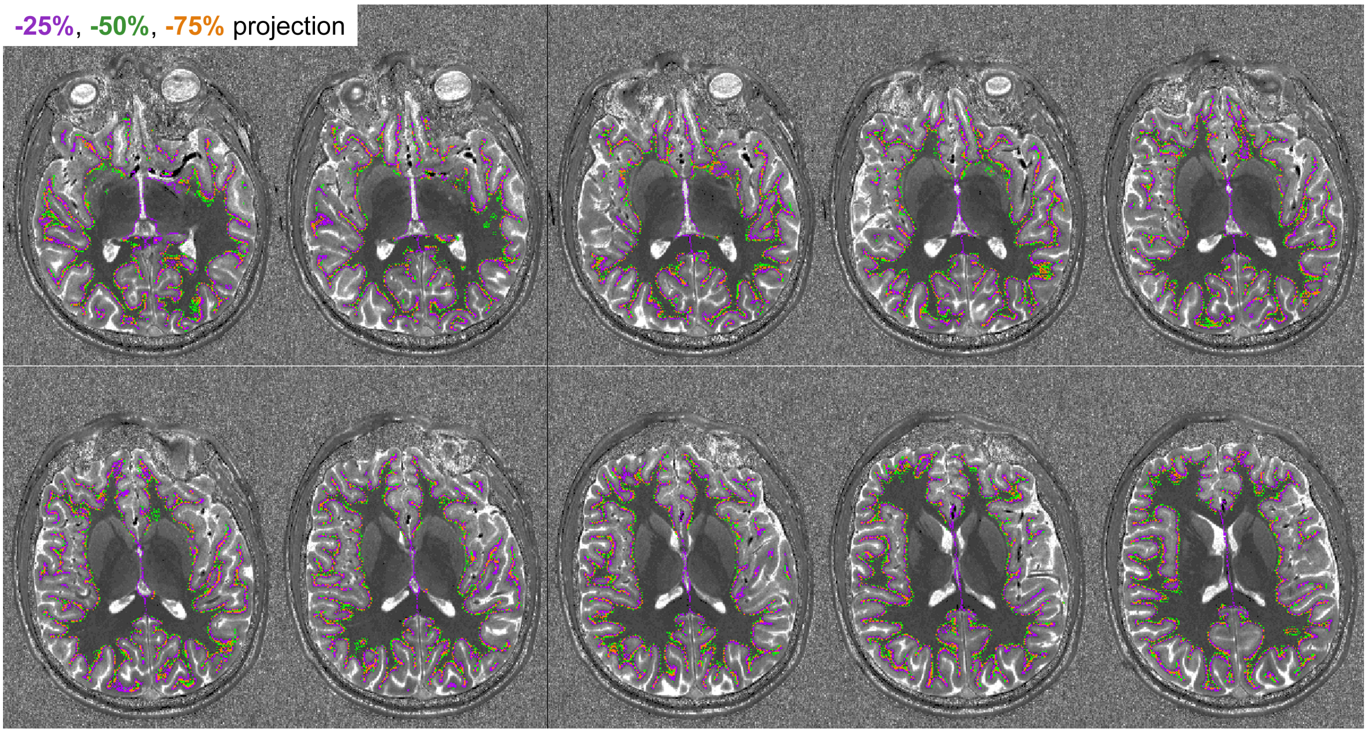

Figure S4. Subcortical white matter sampling points overlaid on the qT1 (=1/qR1) image of a single subject. Purple, green, and orange pixels indicate sampling position in white matter which are shifted inward by the 25%, 50%, and 75% of cortical thickness from the gray and white matter interface, respectively. The sampling points are positioned within the white matter, which appears in dark in the qT1 image.

Using a general linear model (GLM) (Eq. S2) that was used to test AP effect in intracortical myelination, we found an AP effect in the right angular gyrus (AG) (max $T(12) = 6.29$, $p = 0.018$, area =25 mm^2 at the -75 % projection level, peak MNI305-coordinate = [35, -75, 42] mm) as seen in **Figure S5** (upper row).

However, we did not find any differences in subcortical white matter near the right anterior STP, suggesting that the greater cortical myelination in the AP musicians might reflect horizontal, local connections within the cortex rather than vertical, long-range connections.

In addition, we investigated FDT effect in qR1 values in the subcortical white matter using a GLM (Eq. S3) because intersubject variability in FDT might be related to variability in myelination of acoustic radiation (i.e., thalamic projection into primary auditory cortex (PAC). However, we only found a positive correlation between FDT and subcortical myelin in the left orbital sulci (max $T(12) = 9.40$, $p = 0.0002$, area = 254 mm^2 at the -25 % projection; max $T(12) = 8.41$, $p = 0.0158$, area = 48 mm^2 at the -50% projection) as shown in **Figure S5** (lower row) but no effect of FDT in qR1 values beneath the PAC. Significant clusters for the effects of AP and FDT are listed in Table S-III. However, as mentioned earlier, more evidence from other imaging techniques that are better at localizing alteration of white matter myelination is needed.

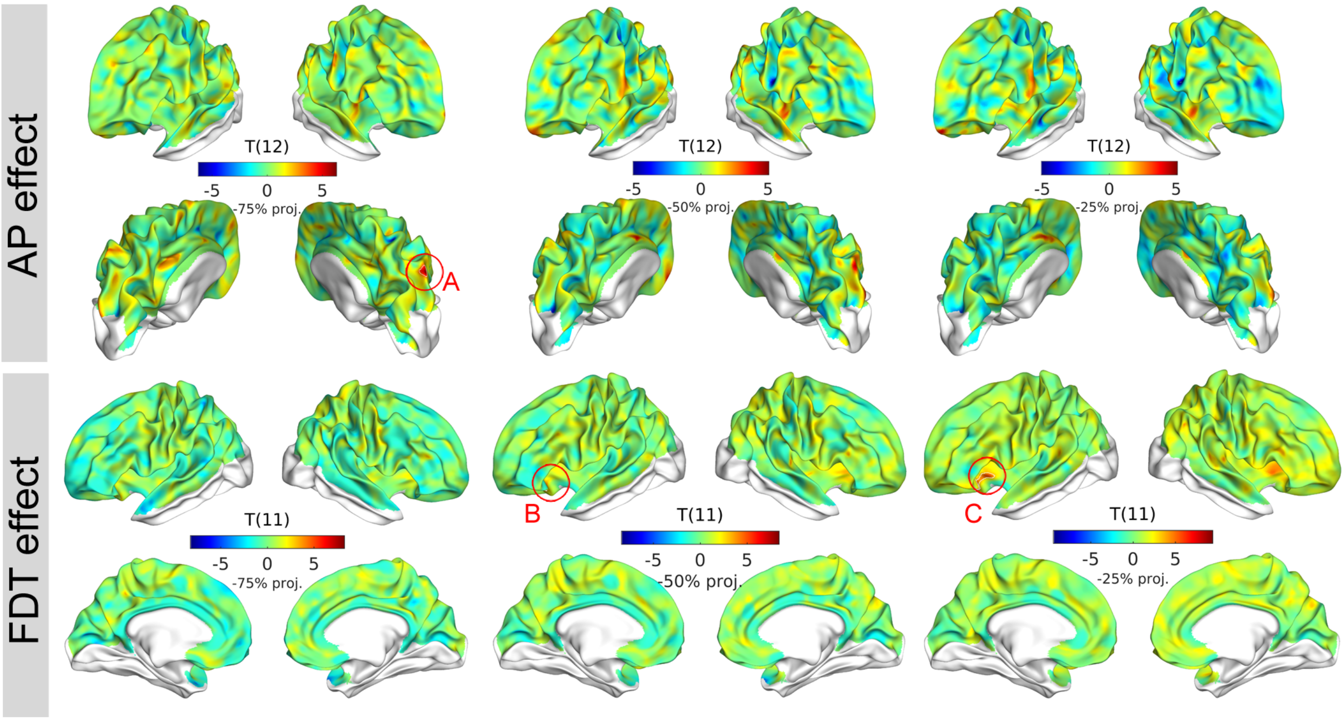

Figure S5. The effect of absolute pitch (AP) and frequency discrimination threshold (FDT) on quantitative longitudinal relaxation rate (qR1) in subcortical white matter. T-statistic maps are given for each projection level. See Table S-III for details of significant clusters.

Table S-III. Significant clusters for the effect of absolute pitch (AP) and frequency discrimination threshold (FDT) in subcortical white matter qR1 values.

Side	Level*	Structure name	T (11)	p-value+	Area (mm2)	MNI-305 (x, y, z mm)

AP effect									
A	Right	-75 %	Right angular gyrus	6.29	0.0323	29.55	34.6	-75.4	42.4
FDT effect									
B	Left	-50 %	Left orbital sulci	8.41	0.0158	47.5	-40.5	31.2	-16.4
C	Left	-25 %	Left orbital sulci	6.01	0.0480	37.6	20.6	-59.1	51.2

* Projection level indicates its sampling point within the subcortical white matter at an inward projection by -75 % (deep), -50 % (middle), or -25 % (superficial) of cortical thickness. + P-values are corrected for multiple comparison correction at cluster level.

Intersubject variability of octave errors

Octave errors are defined as answers with correct pitch chroma, but incorrect pitch height. Because non-absolute pitch (AP) musicians did not give a sufficient number of answers with correct pitch chroma (by definition), we only analyzed octave errors of AP musicians (n=8). We computed the octave error rate (OER), which is a ratio of the number of octave errors to the number of answers with correct pitch chroma, for pure tones and piano tones separately.

As discussed in the Behavioral results subsection in the main text, AP musicians made more octave errors for pure tones compared to piano tones ($T(7) = 5.68$, $p = 0.0007$). The OERs for pure tones were greater than 50% for all AP musicians whereas the OER for piano tones were less than 50% as shown in **Figure S6 A**. In accordance with known dissociation between the acuities in pitch chroma and pitch height (Deutsch, 2013), we did not find any correlation between the OER and the absolute pitch score (APS) ($p = 0.48$, pure tone; $p = 0.56$, piano tone) as in **Figure S6 B, C**. Moreover, no significant correlation between the octave errors and fine-grained relative pitch perception (i.e., FDT) was found ($p = 0.5$ for pure tones; $p = 0.92$ for piano tones) as in **Figure S6 D, E.**

Because the number of AP musicians in the current dataset is limited (n=8), it was difficult to fit a GLM with demographic covariates reasonably (i.e., degrees of freedom = 3). Because age, sex, and ethnicity were still influential also within the AP musicians, we believe controlling the demographic variables is necessary. Further investigation on the octave errors would be possible with a greater dataset of AP musicians in the future.

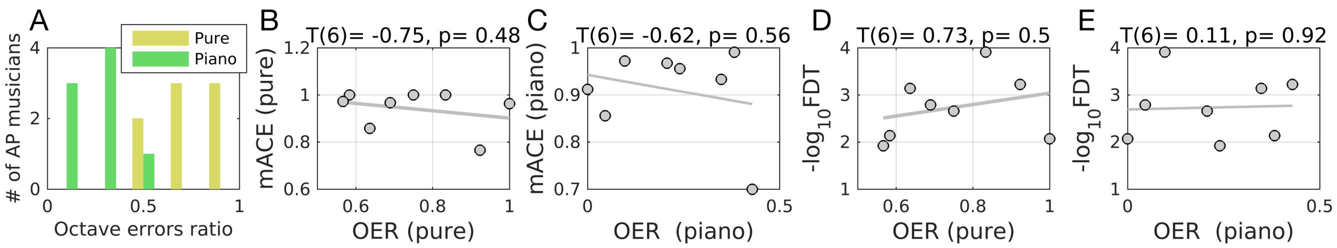

Figure S6. Octave error rate (OER) and its relationship with mean absolute corrected errors (mACE) and frequency discrimination threshold (FDT). Scatterplots for OERs for pure and piano tones (A); OER and mACE for pure tones (B) and piano tones (C); OER for pure tones and FDT (D), and OER for piano tones and FDT (E) are given.

References

Annese J, Pitiot A, Dinov ID, Toga AW. (2004): A myelo-architectonic method for the structural classification of cortical areas. Neuroimage 21(1):15-26.

Ashburner J. (2012): SPM: a history. Neuroimage 62(2):791-800.

Dick F, Tierney AT, Lutti A, Josephs O, Sereno MI, Weiskopf N. (2012): In vivo functional and myeloarchitectonic mapping of human primary auditory areas. J. Neurosci. 32(46):16095-105.

Fischl B. (2012): FreeSurfer. Neuroimage 62(2):774-81.

Jenkinson M, Beckmann CF, Behrens TE, Woolrich MW, Smith SM. (2012): Fsl. Neuroimage 62(2):782-90.

Lutti A, Dick F, Sereno MI, Weiskopf N. (2014): Using high-resolution quantitative mapping of R1 as an index of cortical myelination. Neuroimage 93:176-188.

Marques JP, Kober T, Krueger G, van der Zwaag W, Van de Moortele PF, Gruetter R. (2010): MP2RAGE, a self bias-field corrected sequence for improved segmentation and T1-mapping at high field. Neuroimage 49(2):1271-81.

Micheyl C, Delhommeau K, Perrot X, Oxenham AJ. (2006): Influence of musical and psychoacoustical training on pitch discrimination. Hear. Res. 219(1–2):36--47.

Sereno MI, Lutti A, Weiskopf N, Dick F. (2013): Mapping the human cortical surface by combining quantitative T(1) with retinotopy. Cereb. Cortex 23(9):2261-8.

Shafee R, Buckner RL, Fischl B. (2015): Gray matter myelination of 1555 human brains using partial volume corrected MRI images. Neuroimage 105:473-485.

Waehnert MD, Dinse J, Weiss M, Streicher MN, Waehnert P, Geyer S, Turner R, Bazin PL. (2014): Anatomically motivated modeling of cortical laminae. Neuroimage 93:210-220.
